# A Genome‐Wide Association Screen for Genes Affecting Leaf Trichome Development and Epidermal Metal Accumulation in Arabidopsis

**DOI:** 10.1111/pce.15357

**Published:** 2025-01-15

**Authors:** Radek Bezvoda, Yazmín Mónica Landeo‐Ríos, Zdeňka Kubátová, Eva Kollárová, Ivan Kulich, Wolfgang Busch, Viktor Žárský, Fatima Cvrčková

**Affiliations:** ^1^ Department of Experimental Plant Biology, Faculty of Sciences Charles University Prague Czechia; ^2^ Plant Molecular and Cellular Biology Laboratory, and Integrative Biology Laboratory Salk Institute for Biological Studies La Jolla California USA; ^3^ Gregor Mendel Institute (GMI), Austrian Academy of Sciences Vienna Biocenter (VBC) Vienna Austria; ^4^ Institute of Experimental Botany Czech Academy of Sciences Prague Czechia

**Keywords:** *Arabidopsis thaliana*, BioClim, guard cell, GWAS, metal accumulation, phenotypic variability, trichome

## Abstract

To identify novel genes engaged in plant epidermal development, we characterized the phenotypic variability of rosette leaf epidermis of 310 sequenced *Arabidopsis thaliana* accessions, focusing on trichome shape and distribution, compositional characteristics of the trichome cell wall, and histologically detectable metal ion distribution. Some of these traits correlated with cLimate parameters of our accession's locations of origin, suggesting environmental selection. A novel metal deposition pattern in stomatal guard cells was observed in some accessions. Subsequent GWAS analysis identified 1546 loci with protein sequence‐altering SNPs associated with one or more traits, including 5 genes with previously reported relevant mutant phenotypes and 80 additional genes with known or predicted roles in relevant developmental and cellular processes. Some candidates, including GFS9/TT9, exhibited environmentally correlated allele distribution. Several large gene famiLies, namely DUF674, DUF784, DUF1262, DUF1985, DUF3741, cytochrome P450, receptor‐Like kinases, Cys/His‐rich C1 domain proteins and formins were overrepresented among the candidates for various traits, suggesting epidermal development‐related functions. A possible participation of formins in guard cell metal deposition was supported by observations in available loss of function mutants. Screening of candidate gene lists against the STRING interactome database uncovered several predominantly nuclear protein interaction networks with possible novel roles in epidermal development.

## Summary statement

We performed a GWAS analysis of natural variability of Arabidopsis trichome development and epidermal metal deposition, including its relationship to climate factors. Besides of confirmatory identification of several known genes, we found possible new players contributing to the studied processes.

## 1 Introduction

The plant above‐ground organs epidermis is a sophisticated interface between the plant and its environment, providing mecHanical protection, deterring pathogens and predators and allowing for a controlled gas exchange via stomata. Multiple cell types act in a concerted manner to fulfil these functions, and even subtle disruption of their differentiation may cause observable phenotypic alterations (Zuch et al. 2022). Unconspicuous epidermal defects, well tolerated under laboratory conditions, can indicate perturbation of fundamental cell differentiation or cell morphogenesis processes, as documented, for example, by identification of mutants defective in actin nucleation based on a distorted trichome phenotype (see Szymanski 2005).

The most abundant cell type in the leaf epidermis of the model plant *Arabidopsis thaliana* are the puzzle piece‐shaped pavement cells. Their morphogenesis is driven by microtubule rearrangements that constrict cell surface expansion to generate indentations and allow actin‐dependent intrusion of neighboring cell's lobes. Coordination of these processes involves cell wall‐mediated mecHanical feedback and is orchestrated by a regulatory network including ROP small GTPases, their effectors, locaLized exo‐ and endocytosis and auxin signalLing (see Sapala et al. 2018; Lin and Yang 2020; Liu et al. 2021; Igisch, Miège, and Jaillais 2022). Leaf epidermis also contains stomatal complexes, hydathodes and trichomes, whose patterning is controlled by complex regulatory networks (see Torii 2021). Differentiation of these specialized cell types involves rearrangement of gene expression patterns, well documented in *A. thaliana* for both stomatal guard cells (Zhao et al. 2008; Bates et al. 2012; Xia et al. 2022) and trichomes (Jakoby et al. 2008; Huebbers et al. 2022, 2023).

Trichomes contribute to anti‐herbivore defense by acting as mecHanical deterrents of herbivore feeding; their ecological relevance is well documented (e.g., Sato et al. 2019; Qu, Bonte, and Vandegehuchte 2022). They also engage in detoxification of heavy metals (e.g., Harada et al. 2010; Yaashikaa et al.^2022^) and may sense vibrations and mechanical signals (Zhou et al. 2017; Liu et al. 2017). Development of the branched unicellular *A. thaliana* trichomes is a multi‐stage process (Mathur et al. 1999; Han et al. 2022) involving genome endoreduplication, initiation and expansion of trichome branches, and production of secondary cell wall with a characteristic structure and patterns of callose and metal ion deposition (Kulich et al. 2015, 2018). The ablilty to immobilize metal ions in the trichome cell wall may contribute to detoxication of heavy metals such as excessive zinc (Ricachenevsky et al. 2021) or cadmium (Gao et al. 2021, 2022) by sequestration.

Knowledge regarding *A. thaliana* epidermal development comes mainly from studies on a handful of inbred lineages maintained for decades in the laboratory, such as the widespread Columbia accession. Nevertheless, for some epidermal traits, phenotypic variation among natural Arabidopsis accessions has been attributed to sequence polymorphism in specific genes, such as the *GL1, ETC2* and *MYC1* transcription regulators affecting trichome density (Hauser, Harr, and Schlötterer 2001; Hilscher, Schlötterer, and Hauser 2009; Symonds, Hatlestad, and Lloyd 2011; see also Hauser 2014) or the *STI* gene controlling trichome branch number (Ilgenfritz et al. 2003).

With the onset of high throughput sequencing and availabiLity of genetically characterized resources linked to computational tools, genome‐wide association studies (GWAS) identifying sequence polymorphisms correlated with distinct phenotypic features became a powerful tool for discovering new gene functions. While the GWAS methodology is well established in Arabidopsis (see, e.g., Brachi, Morris, and Borevitz 2011; Slovak et al. 2015), it found only Limited use in studying epidermal development in this species. A screen for host‐side genetic determinants of plant‐associated microbiome variability revealed genomic regions enriched in genes participating in cell wall biogenesis, and somewhat surprisingly, also in trichome branching (Horton et al. 2014). A recent report applied GWAS to identify genes responsible for variation in aerial organ trichome density (Arteaga et al. 2022). Epidermal or trichome characteristics have, however, been addressed by GWAS in non‐model plant species, including commercially important crops. Recent studies focused on trichome distribution patterns in *Brassica napus* (Xuan et al. 2020) and *Aegilops tauschii* (Mahjoob et al. 2022), on cuticular wax deposition in *B. napus* (Jin et al. 2020; Long et al. 2023) and on cotton trichome development (Li et al. 2020; Wang et al. 2021).

Here we report a GWAS for genes associated with phenotypic variability of selected epidermal traits of rosette leaves in 310 sequenced *A. thaliana* accessions from the 1001 Genomes collection (Alonso‐Blanco et al. 2016), focusing on traits related to trichome development, cell wall composition and epidermal metal deposition. Besides identifying genes known or already suspected to affect these characteristics, our results suggest possible roles in epidermal development for several gene families with hitherto uncharacterized or only partly characterized functions.

## 2 Results

### 2.1 Natural Variation of Rosette Leaf Epidermis Traits

We analyzed 310 natural *A. thaliana* accessions, mostly with available detailed location data and climate parameters of their sites of origin (Supporting Information S2: Table 1) to gain insight into the genetic basis of variability in trichome shape, size and density, epidermal autofluorescence and epidermal metal deposition patterns. We recorded 7 continuous quantitative and 11 categorical semiquantitative (ordinate) or qualitative parameters (Table 1; Bezvoda 2023) that exhibited readily noticeable variabiLity among the accession and were amenable to semi‐high‐throughput analysis with good interobserver replicability.

**Table 1:** Epidermal phenotype parameters included in the GWAS screen.

Parameter	Refers to	Variable type	Categorical variable values	Sample preparation
Area	Trichomes	Continuous		Callose staining
Circularity	Trichomes	Continuous		
Solidity	Trichomes	Continuous		
Length	Trichomes	Continuous		
Perimeter	Trichomes	Continuous		
Callose content	Trichomes	Continuous		
Stem length	Trichomes	Continuous		Drying (autofluorescence observed under UV light)
Trichome autofluorescence	Trichomes	Categorical ordinate	0 (none); 0,5; 1; 2; 3 (strongest)	
Epidermis autofluorescence	Epidermis outside trichomes	Categorical ordinate	0 (none); 1; 2; 3; 4; 5 (strongest)	
Autofluorescence colour	Epidermis	Categorical qualitative	0 (none, whitish or colourless); 1 (bluish or brownish hue)	
Trichome density	Epidermis	Categorical ordinate	0 (none); 0.5; 1; 1.5; 2; 2.5; 3; 4 (densest)	
Number of branches	Trichomes	Categorical ordinate	Values roughly correspond to typical (or average) trichome branch number	
Metal ring	Trichomes	Categorical ordinate	0 (no rings); 1 (rings in some trichomes); 2 (rings in most or all trichomes)	Metal staining
Metal gradient	Trichomes	Categorical ordinate	Scale reflects gradient length ‐ 0 (no gradient); 0.5; 1; 1.5; 2; 2.5; 3 (longest)	
Metal base	Trichomes	Categorical ordinate	0 (no staining of trichome base; 1 (in some trichomes); 2 (in most or all trichomes)	
Metal surround	Epidermis around trichome base	Categorical ordinate	0 (no staining around trichome base; 1 (around some trichomes); 2 (around most or all trichomes)	
Metal stomata	Guard cells	Categorical ordinate	0 (no staining; 1 (in some stomata); 2 (in many stomata); 3 (in most or all stomata)	
Shaveproof	Epidermis	Categorical qualitative	0 (trichomes detachable); 1 (shaveproof)	Detachment of trichomes during callose staining

**Figure 1:**
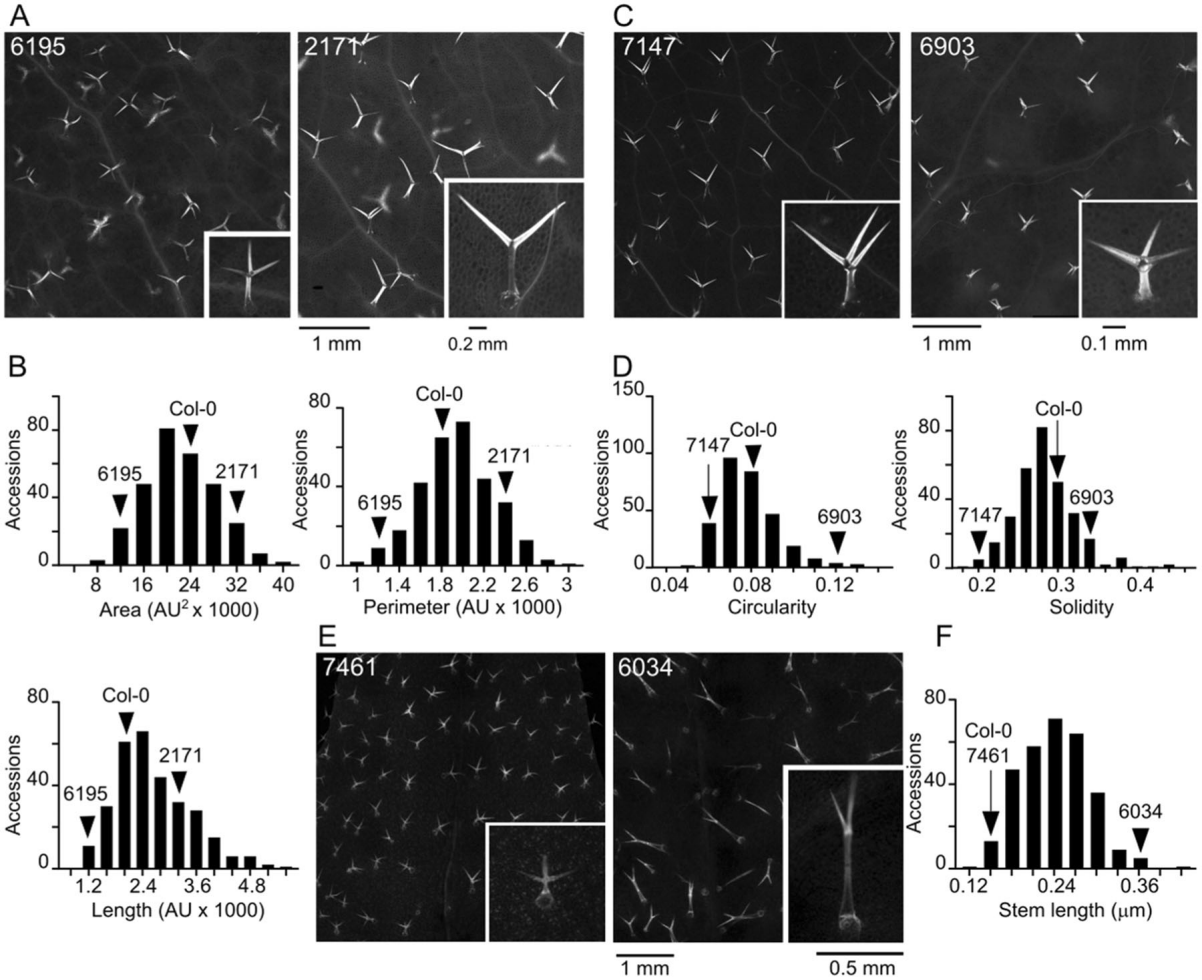
Examples of continuous quantitative parameters of trichome shape. (A) Detailed view of representative leaves of ecotypes with low (6195, TDr‐9) and high (2171, Paw‐26) trichome area, perimeter and length with close‐up of a typical trichome. (B) Histograms showing distribution of area, perimeter and length among analyzed accessions. (C) Detailed view of representative leaves of ecotypes with low (7147, Gie‐0) and high (6903, Bor‐4) trichome circularity and soLidity with close‐up of a typical trichome. (D) Histograms showing distribution of circularity and soLidity among analyzed accessions. (E) Detailed view of representative leaves of ecotypes with low (7461, H55) and high (6034, Hov1‐7) trichome stem length with close‐up of a typical trichome. (F) Histogram presenting distribution of stem length among analyzed accessions. Positions of Listed accessions and Col‐0 in the histograms are marked by arrowheads. AU, arbitrary units.

**Figure 2:**
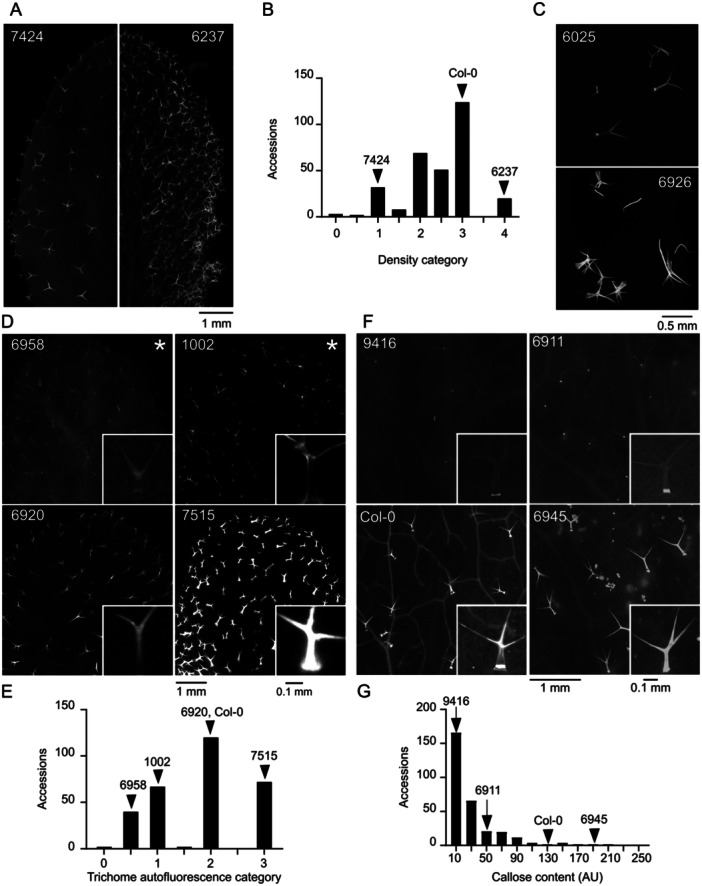
VariabiLity of trichome density, branch number, autofluorescence and callose content. (A) Representative leaf photos of the indicated ecotypes with low (7424, JI‐3) and high (6237, TOM 03) trichome density. (B) Histogram showing distribution of trichome density categories. (C) Typical isolated trichomes of an accession with low (6025, Gro‐3) and high (6926, Kin‐0) branch numbers. (D) Detailed view of representative leaves of ecotypes with different levels of trichome cell wall autofluorescence. (6958, Ra‐0; 1002, Ale‐Stenar‐64‐24; 6920, Got‐22; 7515, RRS‐10), with close‐ups of single typical trichomes. Ecotypes marked by asterisks also represent typical examples of low/absent (1002, Ale‐Stenar‐64‐24) and high (6958, Ra‐0) epidermal autofluorescence outside trichomes. (E) Histogram showing distribution of trichome autofluorescence categories. (F) Detailed view of representative leaves of ecotypes with different levels of callose deposition in trichomes (9416, Ktu‐3; 6911, Cvi‐0; 6909, Col‐0; 6945, Nok‐3), with close‐ups of a single typical trichome. (G) Histogram showing distribution of callose content. Positions of Listed accessions and Col‐0 in the histograms are marked by arrowheads.

The first group of parameters with continuous distribution (Figure 1) describes quantitative aspects of trichome shape, that is, size‐related parameters (area, perimeter, overall length and stem length) and descriptors of shape complexity (circularity and solidity). Other morphological features, namely trichome density (Figure 2A,B) and branch number (Figure 2C), were evaluated semiquantitatively.

**Figure 3:**
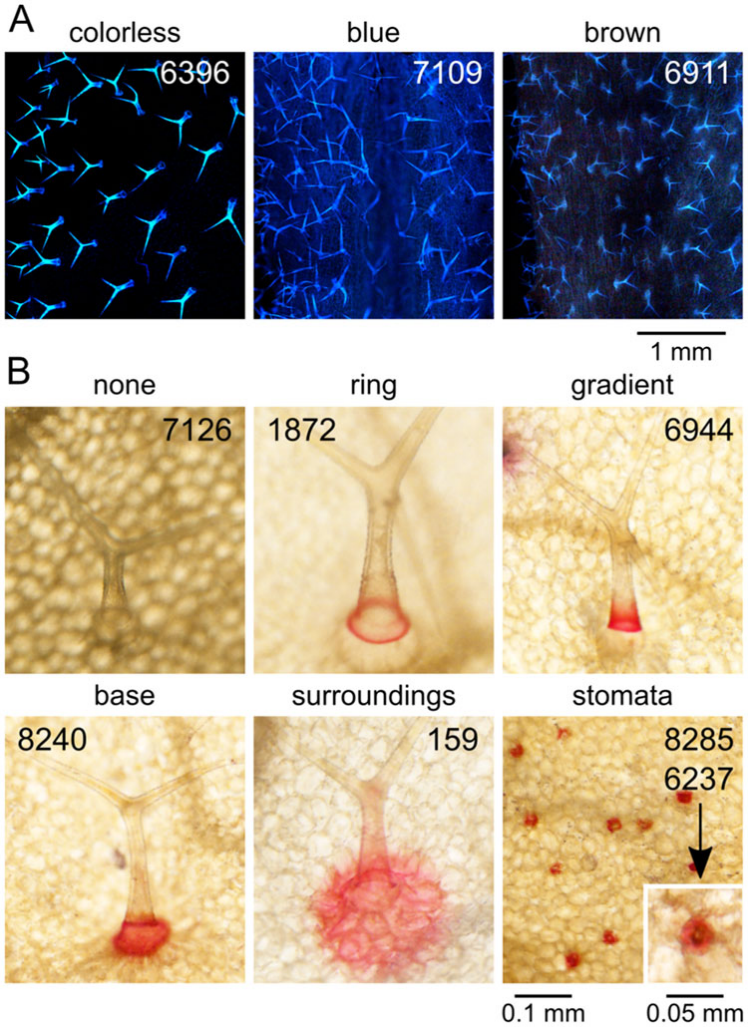
Examples of autofluorescence colours and patterns of trichome and epidermal metal deposition. (A) Representative examples of epidermal autofluorescence colours recorded as ‘colorless’ (no background fluorescence, whitish trichomes—6396, Udul 4‐9) and ‘colored’ (blue—7109, Ema‐1; brownish—6911, Cvi‐0). Contrast of the photographs has been uniformly adjusted using ImageJ's ‘auto’ settings. (B) Images of representative trichomes or epidermis areas for each pattern of metal deposition observed in the screen. Presented pattern is not necessarily the most typical or the only one found for the ecotype shown (none—7126, Es‐0; ring—1872, MNF‐Pot‐75; gradient—6944, NFA‐8; base—8240, Kulturen‐1; surround—159, MAR2‐3; stomata—8285, DraIII‐1; stomata close‐up—6237, TOM 03).

The next group of parameters reflects structural and chemical properties of the trichome cell walls, affecting trichome autofluorescence upon damage, estimated as a semiquantitative categorical variable (Figure 2D,E) and callose content, evaluated semiquantitatively on a continuous scale in a manner capturing also the spatial distribution of callose (Figure 2F,G). Obvious variability in the background epidermal autofluorescence outside trichomes was recorded as a categorical semiquantitative variable (Figure 2D). We introduced another categorical qualitative variable to describe visible differences in the colour of trichome and background fluorescence (Figure 3A).

A third group of categorical semiquantitative parameters captures the spatial distribution of metal ion deposits, as detected by dithizone staining, in trichomes and other epidermal cell types (Figure 3B; for description of parameters see Table 1).

Since we noticed that trichomes of some accessions resist mechanical detachment during staining for callose, we recorded this feature as a qualitative parameter ‘shaveproof’.

**Figure 4:**
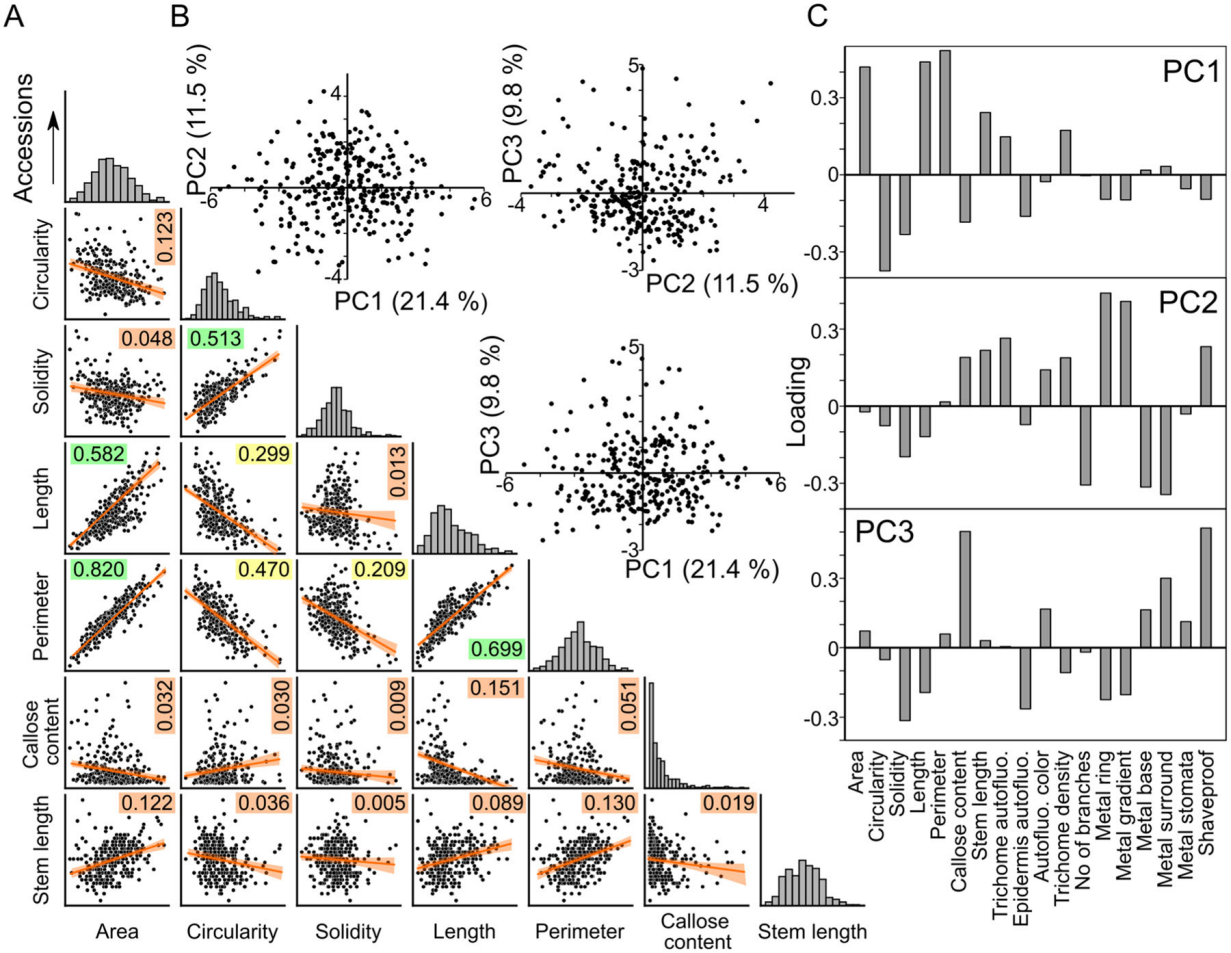
Distribution of epidermal phenotypic variabiLity among accessions. (A) Mutual correlation of the seven continuous‐value quantitative parameters analyzed, with regression lines, confidence intervals and coefficient of determination (*R* ^2^) values indicated. *R* ^2^ values are colour‐coded from green (high, above 0.5) through yellow (intermediate) to orange (below 0.2). For Spearman's correlation coefficients and *p* values see Supporting Information S1: Figure 1. Distribution of values is shown for each parameter at the diagonal of the matrix (scales are arbitrary, for quantitative values see Figures 1B,D,F and 2G). (B) Scatterplot of PCA results reflecting the between‐accession variability, calculated from the complete epidermal phenotype data set (points correspond to individual accessions). Fraction of variance attributable to each PC is shown in parentheses. (C) Loadings of the first three principal components.

All seven continuous variable parameters exhibit smooth distribution with a single roughly symmetrical maximum (Figures 1B,D,F, 2G, 4A), except callose content, where most accessions have very Little if any callose in trichomes, resulting in an asymmetric distribution. A roughly symmetrical single maximum distribution, in some cases noisy, was observed also for the semiquantitative categorical variables of trichome autofluorescence, epidermis autofluorescence, trichome density and trichome branch number. The ‘noisy single maximum distribution’ may be explainable by under‐representation of visually attributed intermediate values, perhaps reflecting observer uncertainty rather than genuine differences. For several other parameters (metal gradient, metal surround, metal stomata) the distribution was asymmetric with most accessions exhibiting low or no signal. Remaining parameters either varied between only two values (autofluorescence colour, shaveproof) or showed a possibly bimodal distribution, such as in the case of metals at trichome base and metal rings (Supporting Information S1: Figure S1).

Not surprisingly, some quantitative descriptors of trichome shape were mutually correlated (Figures 4A and Supporting Information S1: Figure 1). Trichome area, perimeter and overall length, which can all be viewed as measures of trichome size, exhibited strong or very strong positive correlation. Weak to moderate positive correlation with these parameters was found also for trichome stem length. Circularity (proportional to the ratio of an object's area and squared perimeter) and soLidity (ratio of the area of the examined object and its convex hull), which both achieve higher values for less intricate shapes, were mutually moderately positively correlated, and negatively correlated with the size‐related parameters. While there were some cases of non‐negligible correlation among the categorical variables, or between categorical and continuous ones, such correlations were generally weak except of moderate positive correlation between the frequency of metal rings and intensity of metal gradients at the trichome stems, as well as moderate positive correlation between strength of mechanical attachment (the ‘shaveproof’ trait) and trichome callose content, which was also moderately negatively correlated with trichome length. Remarkably, all the strong or moderate correlations were statistically significant (Supporting Information S1: Figure 1).

For a subset of traits including representatives of the mutually correlated trait groups, heritability was estimated from data collected separately for individual plants (Supporting Information S2: Table 2). The broad sense heritability values, ranging between 0.58 and 0.94, indicate a strong genetic contribution to the observed variability (Supporting Information S2: Table 3).

Principal component analysis (PCA) of the 18 recorded parameters did not reveal any obvious clustering of accessions when considering the first three principal components (PCs), which, however, cumulatively explained only less than 43% of total variability (Figure 4B). Analysis of PC loadings indicated that PC1 is positively correlated with the mutually correlated parameters reflecting trichome size, and negatively with circularity and solidity, PC2 correlates either positively or negatively with specific patterns of metal staining, and PC3 mostly reflects callose content and strength of mechanical attachment—that is, two parameters related to cell wall structure (Figure 4C). The PCA results thus support the overall pattern of continuous variability of traits addressed in our study, and also suggest that trichome shape, metal staining and cell wall organization might be determined by distinct sets of genes.

**Figure 5:**
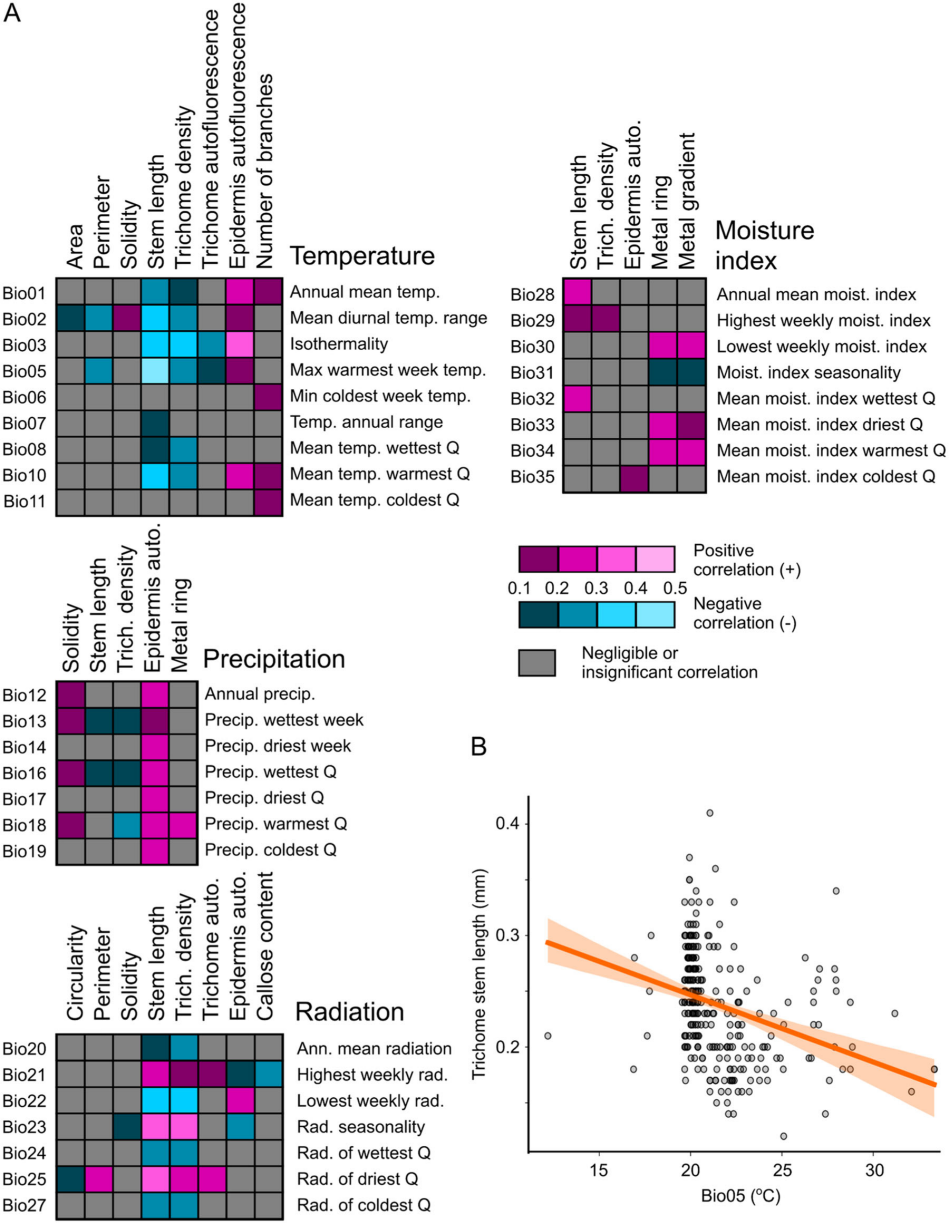
Correlations of epidermal traits and cLimatic variables. (A) Heatmaps summarizing all statistically significant non‐negLigible correlations among epidermal traits and BioClim variables of the site of origin, grouped according to underlying environmental factors and colour‐coded according to the Spearman's correlation coefficient values. For a complete list of combinations, correlation coefficient values and *p* values see Supporting Information S2: Table 4. (B) Correlation of trichome stem length and the maximum temperature of the warmest week at the site of origin (variable Bio05). The coefficient of determination (*R* ^2^) value was 0.204, that is, in the intermediate range.

We next examined possible relationships between the 18 phenotypic traits determined in our study and 35 standardized climate variables of the sites of origin of our Arabidopsis accessions, obtained from the CliMond database (Kriticos et al. 2012). Out of the 630 possible trait and cLimatic variable combinations, only 81 exhibited statistically significant non‐negligible positive or negative correlation, typically in the weak range (Supporting Information S2: Table 4, Figure 5A). The only exception was a negative correlation between trichome stem length and the maximum temperature of the warmest week (the Bio05 variable), which fell into the moderate range (*sensu* Schober, Boer, and Schwarte 2018; Figure 5B). The biological interpretation of this observation is not immediately obvious.

### 2.2 GWAS Analysis Identifies Loci Associated With Some Epidermal Traits

The phenotypic data were subjected to association analysis using the publicly available GWAPP GWAS platform (Seren et al. 2012) as described in Materials and Methods, aiming first towards identification of all single nucleotide polymorphisms (SNPs) within the transcribed section of genomic DNA, including the CDS, introns and the 5ʹ and 3ʹ UTRs. The numbers of identified significantly associated loci varied among traits on the scale from zero to several hundred, with trichome stem length and guard cell metal accumulation yielding the most candidates (Tables 2 and Supporting Information S2: Table 5). No significant SNPs were found for trichome area, metal ring, metal staining of trichome bases, and resistance towards mechanical detachment, and only one or a few presumably silent polymorphisms (i.e., synonymous mutations or SNPs located in an intron or in untranslated mRNA regions) were identified for overall trichome length, number of branches and the presence of a metal gradient. These traits were therefore not included in subsequent analyses.

**Table 2:** Numbers of loci and polymorphism types identified as significantly associated with individual parameters.

Parameter	Start/stop change	Missense	UTR or intron	Synonymous
Area	0	0	0	0
Circularity	0	44	86	25
Solidity	0	33	66	16
Length	0	0	1	0
Perimeter	0	6	15	3
Callose content	0	1	2	1
Stem length	26	657	1197	574
Trichome autofluorescence	0	21	39	12
Epidermis autofluorescence	0	11	16	4
Autofluorescence colour	1	52	95	35
Trichome density	2	216	357	182
Number of branches	0	0	1	0
Metal ring	0	0	0	0
Metal gradient	0	0	2	1
Metal base	0	0	0	0
Metal surround	0	16	30	17
Metal stomata	27	627	970	425
Shaveproof	0	0	0	0

The distribution of SNP types for individual traits with multiple SNPs exhibits some differences, although they are not major (Supporting Information S1: Figure 2). In particular, SNPs altering CDS length were found only for traits with overall large SNP numbers, reflecting low probability of polymorphisms affecting start and stop codons. Subsequently, we focused only on loci with SNPs changing the predicted protein product sequence, in most cases due to a missense mutation, and treated all such SNPs as a single category. The full list of genes affected by such SNPs for each trait is provided in Supporting Information S2: Table 6. In total, 1547 different loci exhibited association with at least one of the studied traits, corresponding to ~5.6% of all Arabidopsis genes.

**Figure 6:**
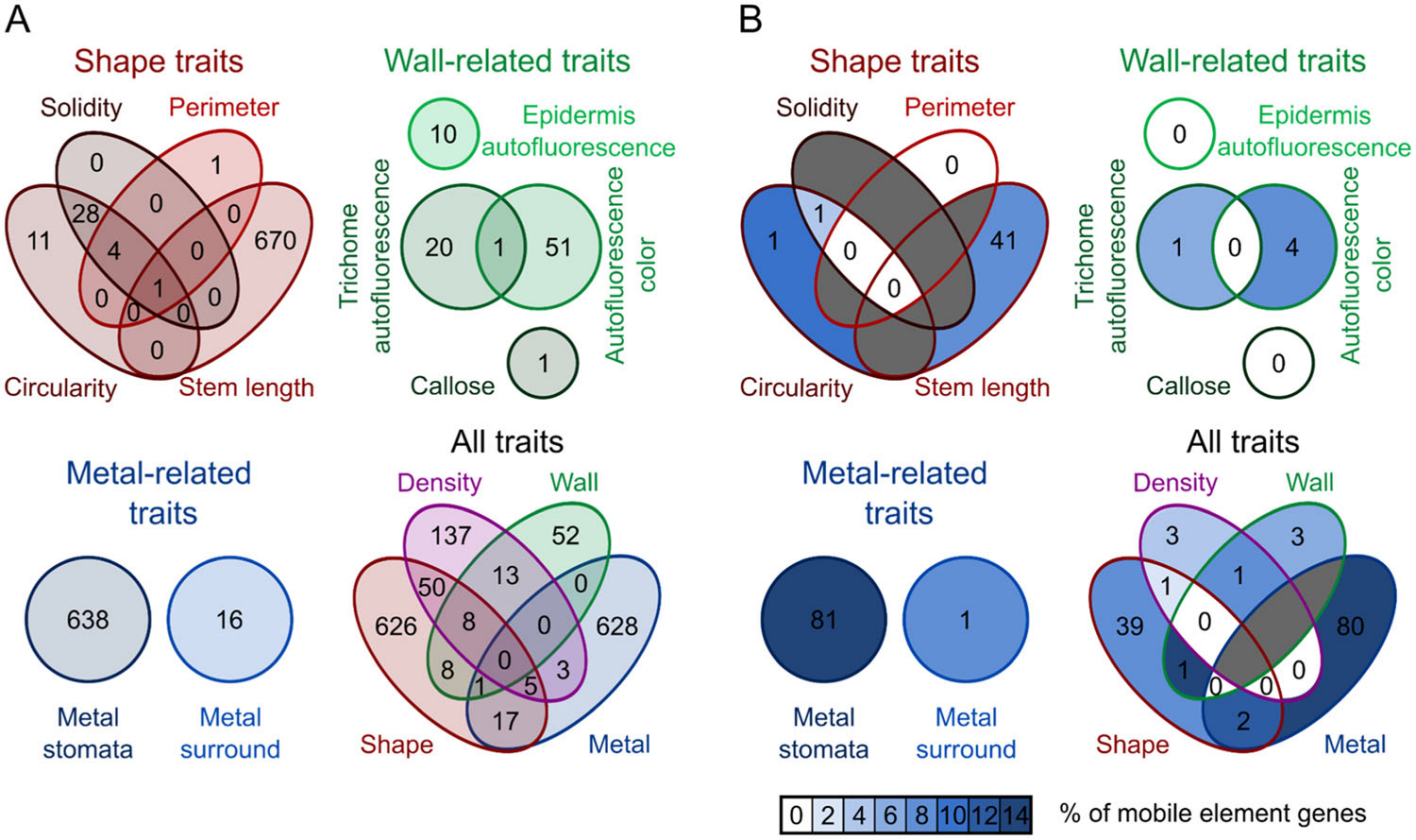
Unique and shared SNPs among traits and trait groups. Venn diagrams showing numbers of loci affected by ORF‐changing SNPs associated with individual traits and trait groups and/or shared by multiple traits or trait groups. (A) All ORF‐changing SNPs. (B) ORF‐changing SNPs in loci contained within transposons. Fields are colour‐coded according to proportion of transposon‐derived loci among all loci detected for the given trait combination. Dark grey fields indicate trait combinations with no associated SNPs detected.

For further analyses, we divided the traits with significantly associated SNPs into four groups: those affecting trichome shape (circularity, soLidity, perimeter and stem length), trichome distribution on the surface of the leaf (i.e., trichome density), wall composition‐related parameters (epidermis and trichome autofluorescence, autofluorescence colour, callose content) and metal accumulation (metal staining of stomata and trichome surroundings). While some loci associated with several of the mutually correlated cell shape traits, there was only a very Limited, if any, overlap between candidate lists for traits from the wall composition‐related and metal accumulation‐related groups. However, polymorphisms in some loci were identified as associating with traits from more than one category (Figure 6A, Supporting Information S2: Table 7).

Assuming that SNPs in transposon‐borne genes are unLikely to cause phenotypic differences, we examined what fraction of our candidates corresponds to CDSs from mobile genetic elements to gain insights into the extent of non‐specific background. The fraction of transposon‐derived loci was significantly depleted compared to whole genome values for the sum of all GWAS candidates, as well as for those associated with shape‐related traits or trichome density. However, while depletion of transposon genes was also noticeable for wall‐related traits, it was only marginally significant, possibly due to a relatively low number of loci, and practically no depletion was observed for metal‐related traits (Table 3). Sets of genes associated with multiple loci usually contained fewer transposon‐derived loci than those found on the basis of a single trait (Figure 6B).

**Table 3:** Distribution of mobile element‐derived genes among candidate loci associated with ORF‐changing SNPs.

Trait group	All loci	Transposon loci	Fraction from transposons	*p* value
Whole genome (Araport)	27655	3879	14%	N.A.
All traits	1547	129	8%	< 0.0001
Shape traits	715	43	6%	< 0.0001
Density	216	5	2%	< 0.0001
Wall‐related traits	82	6	7%	0.10
Metal‐related traits	654	82	13%	0.25

*Note: p* values indicate significance of the transposon‐derived locus depletion compared to the whole genome data set (pairwise *χ* ^2^ test with Benjamini–Hochberg correction for multiplicity).

Abbreviation: N.A., not available.

### 2.3 Multiple GWAS Candidates Encode Proteins With Known or Suspected Epidermal Roles

Next, we examined the traits related to trichome development (i.e., the shape traits, density and the cell wall parameters that largely reflect trichome properties) for enrichment of genes abundantly expressed in trichomes either at the mRNA (Jakoby et al. 2008) or protein (Huebbers et al. 2022) levels. While we did not detect significant enrichment or depletion of these genes in our candidates lists, we identified multiple genes associated with trichome related traits that were abundantly expressed in trichomes, consistent with their participation in trichome development. In some cases, available annotations suggest function in membrane trafficking, biogenesis of cell wall components or turgor generation and maintenance. This might point to possible mechanisms whereby variation in the responsible loci may affect cell growth or cell wall development and generate the observed phenotypic diversity, either by direct modification of trichome morphogenesis or by modulating epidermal cell expansion and hence trichome density (Table 4). Prompted by the finding of several genes engaged in cuticle or epidermal wax biosynthesis, that is, traits often linked to trichome development (see Berhin, Nawrath, and Hachez 2022) among these loci, we examined the rest of our candidates for overlap with a list of 87 putative cuticle and wax biosynthesis genes identified on the basis of their known activities and/or expression patterns (Suh et al. 2005). However, we did not find any additional loci in this manner beyond two genes that were also highly expressed in trichomes—namely AT1G01600/CYP86A4 and AT1G76690/OPR2 (see Table 4).

**Table 4:** Candidate loci associated with trichome development traits and highly expressed in trichomes, with phenotypic effects of identified substitutions indicated.

Locus (gene name)	Screen	Description	Trait	Allele (effect)
AT1G01600 (CYP86A4)	T	Member of the CYP86A subfamily of cytochrome p450 genes. Involved in cutin and wax biogenesis (Camoirano et al. 2020)	Stem length	V341I (up)
			Density	A476V (down)
			Trichome autofluorescence	A476V (down)
AT1G08920 (ESL1)	T	ERD (early response to dehydration) six‐like 1; ESL1, a transporter for monosaccharides	Stem length	L118F (up)
			Density	L118F (up)
AT1G12550 (HPR3)	P	Glyoxylate/hydroxypyruvate reductase HPR3. Transcript restricted to trichomes (Xu et al. 2018)	Stem length	S135A (up)
AT1G12570 (HTH‐like)	P	Glucose‐methanol‐choline (GMC) oxidoreductase family protein. Ortholog of maize IPE1 participating in pollen exine development. Involved in cutin and wax biogenesis (Kannangara et al. 2007).	Stem length^a^	K138R (up) N187K (up)
			Density	D77G^b^, S78A^b^ (up)
AT1G15670 (KFB1, KMD2)	T	F‐box/Kelch‐repeat protein. Member of the KISS ME DEADLY (KMD) family, that targets type‐B ARR proteins for degradation and is involved in negative regulation of the cytokinin response. Also known as KFB1, a member of a group of Kelch repeat F‐box proteins negatively regulating phenylpropanoid biosynthesis.	Stem length	E37A (up)
AT1G26400 (FOX3, ATBBE5)	P	Berberine bridge enzyme‐like 5. Possible role in defense, related proteins participate in wall modification (Eggers et al. 2021).	Stem length	I16V^b^, L39P^b^ (up)
AT1G53210 (NCL)	P	Sodium/calcium exchanger NCL	Stem length	G420D (down)
AT1G76690 (OPR2)	P	12‐oxophytodienoic acid reductase with possible role in cutin or wax synthesis (Suh et al. 2005)	Stem length	A90T (down)
AT1G78920 (AVPL1)	P	Pyrophosphate‐energized membrane proton pump 2. Located at Golgi, strong expression in trichomes confirmed using a GUS reporter (Mitsuda et al. 2001).	Stem length	N16S (up)
AT2G20240 (TRM17)	P	GPI‐anchored adhesin‐like protein, putative (DUF3741)	Stem length	S157P, D576E^b^, A671T^b^ (up)
AT2G20990 (SYTA, SYT1)	P	Synaptotagmin A. Regulates endosome recycling at the plasma membrane, but not secretory traffic. Strong expression in trichomes confirmed using a GUS reporter (Schapire et al. 2008).	Stem length	H357Q (up)
AT2G21410 (VHA‐A2)	P	V‐type proton ATPase subunit a2. Required for endocytic and secretory trafficking (Dettmer et al. 2006).	Stem length	I519V^b^, I762V (up)
AT2G21870 (MGP1, PHI1)	P	Probable ATP synthase 24 kDa subunit, mitochondrial. Essential for pollen formation.	Stem length	K207M (up)
AT2G35860 (FLA16)	P	FascicLin‐Like arabinogalactan protein. Mutant has reduced cell size in some cell types in the stem (Liu et al. 2020).	Density	K115T (down)
AT3G28500 (RPP2C, P2X)	P	60S acidic ribosomal protein P2‐3	Density	G82A (down)
AT3G48000 (ALDH2B4)	P	Aldehyde dehydrogenase family 2 member B4, mitochondrial. May participate in biosynthesis of ferulic acid and sinapic acid, modulating cell wall strength (Brocker et al. 2013).	Stem length	N63D (up)
AT3G59010 (PME35, PME61)	P	Pectin methyl esterase, activity controls cell wall mechanic strength; identified in a trichome transcriptome study (Wienkoop et al. 2004)	Density	V291I (down)
AT4G02930 (TUFA)	P	Elongation factor Tu, mitochondrial	Stem length	V29I (down)
AT4G24840 (COG2)	P	OLigomeric Golgi complex subunit‐like protein containing the COG2 domain, part of the membrane trafficking machinery (see Vukašinović and Žárský 2016)	Stem length	L652F (down)
AT5G28220	P	Protein prenylyltransferase (uncharacterized)	Stem length	L190F (down)
AT5G63910 (FCLY)	P	Farnesylcysteine lyase (EC 1.8.3.5) involved in a salvage/detoxification of farnesylcysteine residues liberated during the degradation of prenylated proteins (Crowell et al. 2007)	Density	G88S (down)

*Note:* Unless additional references are provided, annotation is derived from Araport 11 or the TAIR description (Reiser et al. 2024). Substitutions and effect directions refer to the Col‐0 sequence and phenotype.

Abbreviations: P, proteome; T, transcriptome.

a The listed minor alleles affecting this parameter are often present together.

b Not documented in some of the ecotypes carrying the other listed substitution because of sequencing gaps.

Because the loci associated with metal accumulation‐related traits mainly reflect stomatal metal deposition, we examined the metal‐related candidate list for overlap with a published set of 63 genes upregulated in guard cells (Leonhardt et al. 2004), as well as a list of 1508 proteins repeatedly identified in guard cell proteome (Zhao et al. 2008). We found no overlap between our candidate list and the list of genes highly transcribed in guard cells, but identified 21 loci from the proteome set, all of them associated with metal accumulation in the guard cells and some encoding proteins engaged in ion or other solute transport, membrane trafficking, cell wall biogenesis or gene expression, that is, processes possibly relevant for the observed trait (Table 5). However, there was no significant enrichment of guard cell‐expressed genes among our candidates, similar to the trichome case.

**Table 5:** Candidate loci associated with stomatal metal accumulation and expressed in the guard cell proteome.

Locus (gene name)	Description	Allele
AT1G31850	S‐adenosyl‐L‐methionine‐dependent methyltransferases superfamily protein; probable methyltransferase PMT20; found in a Golgi complex proteome study (Parsons et al. 2012)	V113A
AT1G42550 (PMI1)	Protein PLASTID MOVEMENT IMPAIRED 1, a plant‐specific protein of unknown function conserved among angiosperms	G460A
AT1G59820 (ALA3)	PhosphoLipid‐transporting ATPase (phosphoLipid translocase) involved in secretory vesicle formation from trans‐Golgi	E647K
AT1G59900 (E1 ALPHA)	Pyruvate dehydrogenase E1 component subunit alpha‐1, mitochondrial	S361P
AT1G67490 (GCS1, KNF, KNOPF)	Mannosyl‐oLigosaccharide glucosidase GCS1; an alpha‐glucosidase I enzyme that catalyzes the first step in N‐linked glycan processing. Localized to the endoplasmic reticulum.	D567E
AT3G10670 (ABCI6, NAP7)	ABC transporter I family member 6, chloroplastic. Plastidic SufC‐Like ATP‐binding cassette/ATPase essential for Arabidopsis embryogenesis. Involved in the biogenesis and/or repair of oxidatively damaged Fe–S clusters.	L42V
AT3G13460 (ECT2)	Evolutionarily conserved C‐terminal region 1; regulates the mRNA levels of the proteasome regulator PTRE1 and of several 20S proteasome subunits, resulting in enhanced 26S proteasome activity	T210P
AT3G13772 (TMN7)	Transmembrane 9 superfamily member, localized in the secretory pathway. Overexpression of this protein in yeast alters copper and zinc homeostasis (Hegelund et al. 2010).	N565S
AT3G48000 (ALDH2B4, ALDH2, ALDH2A)	Aldehyde dehydrogenase family 2 member B4, mitochondrial	A267T
AT4G13360	3‐hydroxyisobutyryl‐CoA hydrolase‐Like protein 3, mitochondrial; ATP‐dependent caseinolytic (Clp) protease/crotonase family protein	T395A
AT4G18290 (KAT2)	Member of the Shaker family potassium ion (K+) channel. Critical to stomatal opening induced by blue light and to circadian rhythm of stomatal opening. Involved in plant development in response to high light intensity.	L170H
AT4G18670 (LRX5)	Leucine‐rich repeat extensin‐like protein 5, involved in cell wall biogenesis and organization. Interacts with several members of the RALF family of ligand peptides.	P507T P813L
AT4G20400 (JMJ14)	Histone H3K4 demethylase repressing floral transition	P480L
AT4G21180(ERDJ2B)	DnaJ domain protein localized in ER membrane	T67A
AT5G35430(NOT10)	Tetratricopeptide repeat (TPR)‐like superfamily protein, found also in stress granules	W680C
AT5G39040(ABCB27, ALS1, TAP2)	Aluminum sensitive 1, ABC transporter B family member 27; a member of TAP subfamily of ABC transporters that is located in the vacuole. Mutants are hypersensitive to aluminum. May be important for intracellular movement of some substrate, possibly chelated Al, as part of a mechanism of aluminum sequestration.	V266I
AT5G43130(TAF4B)	Transcription initiation factor TFIID subunit 4b	Q120K
AT5G52470(MED36B, FBR1, FIB1, SKIP7)	Probable mediator of RNA polymerase II transcription subunit 36b; encodes a fibrillarin, a key nucleolar protein in eukaryotes which associates with box C/D small nucleolar RNAs (snoRNAs) directing 2'‐O‐ribose methylation of the rRNA	L133V
AT5G55040(BRD13)	DNA‐binding bromodomain‐containing protein; interacts with core SWI/SNF complex components. Identified as a specific subunit of BRM‐associated SWI/SNF (BAS) complexes.	S256A
AT5G58100(IEF7)	Encodes a membrane protein involved in pollen nexine and sexine development	N358H
AT5G58140(PHOT2)	Phototropin‐2; membrane‐bound protein serine/threonine kinase that functions as blue light photoreceptor in redundancy with PHO1. Involved in stomatal opening, chloroplast movement and phototropism.	R898G

*Note:* Unless additional references are provided, annotation is derived from Araport 11 or the TAIR description (Reiser et al. 2024). Substitutions refer to the Col‐0 sequence; all Listed alleles exhibited increased guard cell metal deposition compared to Col‐0.

To further verify the performance of our GWAS screen, we inspected the candidate gene lists for genes previously reported to affect the studied traits. While such a search cannot be exhaustive, we found at least five previously characterized relevant genes (Table 6), including loci coding for two cytoskeletal proteins CLASP and SCAR/DIS3, as well as the EXO84B gene, encoding a subunit of the exocyst complex that regulates targeting of membrane vesicles. All three genes exhibit mutant phenotypes involving trichome shape alterations, and all associated with trichome shape traits. In addition, the gene for another exocyst subunit, EXO70H4, documented to participate in the deposition of metals in the trichome cell wall, was picked up as associating with guard cell metal deposits. The fifth gene, GFS9/TT9, is known to affect cell wall autofluorescence and was found in association with trichome density and autofluorescence traits. Notably, five SNPs affecting this gene often occurred together, constituting a quintuple substitution minor allele associated with reduced trichome density and decreased autofluorescence.

**Table 6:** Candidate loci that were previously characterized as participating in the development of relevant epidermal traits, with phenotypic effects of identified substitutions indicated.

Locus (gene name)	Description	Trait(s)	Allele (effect)
AT2G20190 (CLASP)	CLiP‐associated protein; a microtubule‐associated protein involved in both cell division and cell expansion. It likely promotes microtubule stability. Loss of function mutants have decreased trichome branch number (Pietra et al. 2013).	Stem length	M1399L (up)
AT2G38440 (DIS3, ITB1, SCAR2, WAVE4)	Subunit of the WAVE complex, required for activation of ARP2/3 actin nucleation complex. Mutations cause defects in both the actin and microtubule cytoskeletons that result in aberrant epidermal cell expansion. Loss of function mutants exhibit a distorted trichome phenotype (Basu et al. 2005).	Circularity	I1265V (down)
		Solidity	I1265V (down)
		Density	I1265V (down)
AT3G09520 (EXO70H4)	A member of the EXO70 gene family of putative exocyst subunits, conserved in land plants. Involved in trichome cell wall maturation, required for deposition of heavy metals in the cell wall ([Kulich et al. 2015], though guard cell localization was not specifically observed).	Metal stomata	S93N (up)
AT3G28430 (GFS9, TT9)	Peripheral membrane protein localized at the Golgi apparatus that is involved in membrane trafficking, vacuole development and flavonoid accumulation. Loss of function mutants exhibit abnormal autofluorescence, though trichomes were not specifically examined (Ichino et al. 2014).	Density	S573A, S626A, Y667H^a^, A670V^a^, D671E^a^ (down)
		Trichome autofluorescence.	S573A, S626A, Y667H^a^, A670V^a^, D671E^a^ (down)
AT5G49830 (EXO84B)	Exocyst complex component 84B; involved in tethering vesicles to the plasma membrane. Interacts with Exo70H4 that controls trichome cell wall development; loss of function mutant has multiple defects including in trichome morphogenesis (Kulich et al. 2015; Fendrych et al. 2010).	Circularity	E123G (down)
		Solidity	E123G (down)

*Note:* Substitutions and effect directions refer to the Col‐0 sequence and phenotype.

^a^ Not documented in some of the ecotypes carrying the other listed substitution because of sequencing gaps.

Additional 80 genes with previously reported roles in cytoskeletal organization, membrane trafficking, cell wall biogenesis, tissue patterning or other relevant functions were also found among the candidates, including several genes affecting aspects of epidermal development unrelated to traits addressed in our study (Supporting Information S2: Table 8).

These findings suggest that, despite the lower efficiency of the metal trait‐associated gene search, as evident from the lack of transposon gene depletion, the set of candidates found in our screen contains at least a sizeable proportion of genes biologically relevant in the context of leaf epidermis differentiation.

### 2.4 Some GWAS Candidates Exhibit Environmentally Correlated Allele Distribution

To obtain clues to the mechanisms underlying the observed correlation between trichome stem length and the maximum warmest week temperature of the accession's location of origin (the Bio05 variable), we further examined the set of genes exhibiting SNPs associated with trichome stem length. Since the full List of over 600 stem length‐associated candidates was too large for a detailed study, we focused on the 18 genes that were at the same time abundantly expressed in trichomes or encoded proteins with known roles in trichome development (see Tables 4 and 6).

**Figure 7:**
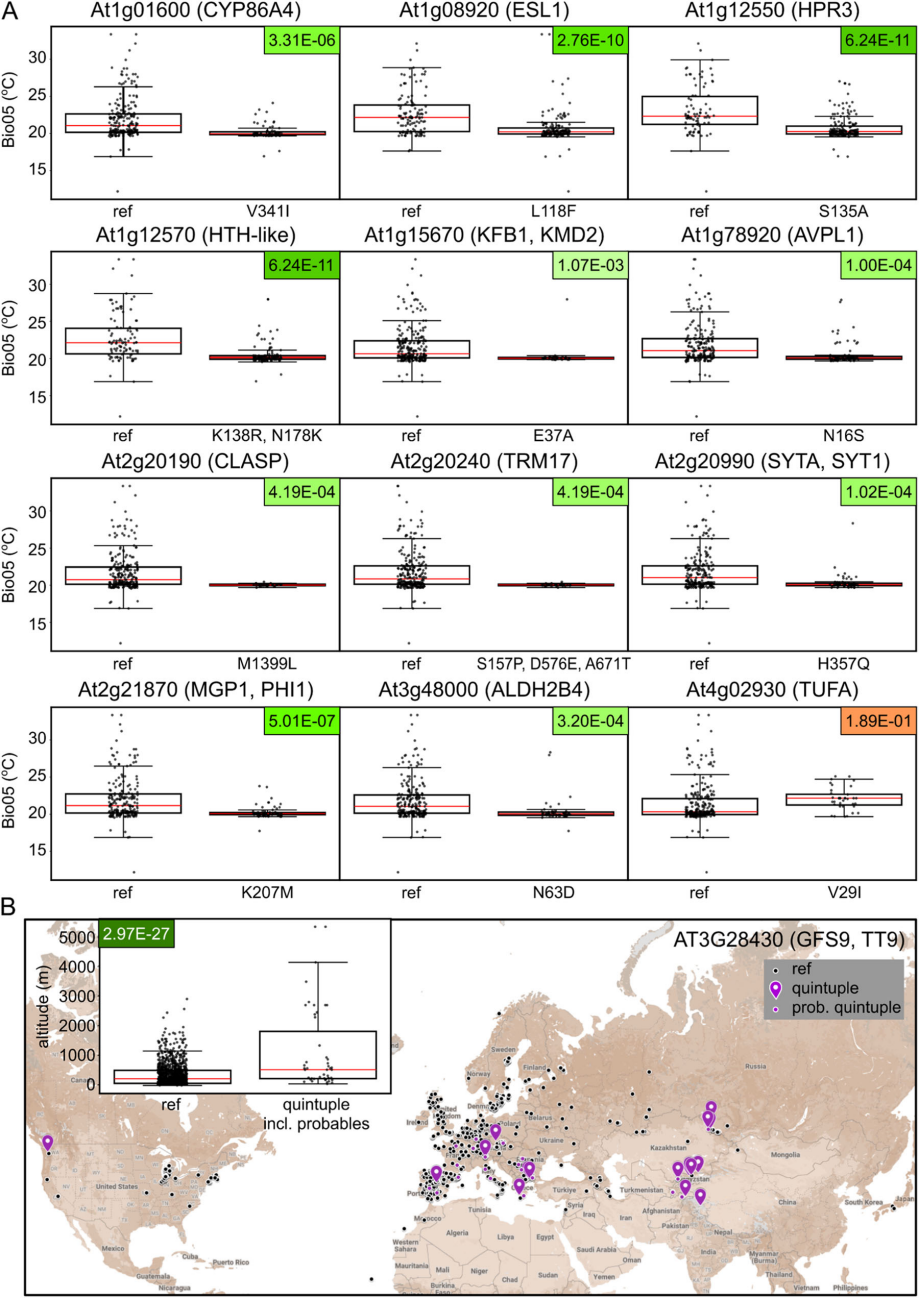
Environmentally correlated allele distribution of selected loci. (A) Distribution of the Bio05 cLimatic variable values for reference and minor alleles of selected genes linked to the trichome stem length trait among our experimentally characterized Arabidopsis accessions. (B) Geographic distribution of populations from the whole Ensembl data set carrying the reference versus quintuple minor (S573A, S626A, Y667H, A670V, D671E) allele of GFS9/TT9. Accessions where some of the SNPs characteristic for the quintuple allele are missing due to sequencing gaps are categorized as ‘probable quintuple’. Inset—distribution of the altitude parameter among accessions with the reference or quintuple minor allele of GFS9/TT9 (including the probable quintuple substitution accessions). FDR‐corrected *p* values for the observed differences are colour‐coded green for highly significant, 0 < *p* ≤ 0.01 (with darker colour corresponding to lower P) or orange for not significant (*p* > 0.05). Red lines indicate median values.

For twelve of these genes, at least 30 studied genotypes (i.e., a tenth of our accessions set) harboured the minor allele. The distribution of Bio05 values for these loci in all but one case exhibited a statistically significant difference between the major and minor alleles, indicating an environmentally correlated bias in allele distribution (Figure 7A), which might suggest natural selection against the minor alleles in hot habitats. Among the affected genes were loci encoding the microtubule‐associated protein CLASP, known to have pleiotropic roles in cell expansion and cell division (Ambrose et al. 2007; Pietra et al. 2013), and SYT1, coding for a synaptotagmin engaged in processes related to stress tolerance and immunity towards pathogens (Schapire et al. 2008; Yamazaki et al. 2008; Levy, Zheng, and Lazarowitz 2015; Kim et al. 2016). It this thus conceivable that the selection was operating on some function(s) of these genes unrelated to epidermal morphogenesis.

For all genes with significant environmentally correlated allele distribution, populations harbouring minor alleles originated from multiple sites, i.e. there was no evidence of spatial restriction due to a founder effect. Even variants only found in Scandinavian accessions occured at two or more geographically distant sites (Supporting Information S1: Figure 3). This further supports the relevance of environmental factors and possible natural selection.

Since the GFS9/TT9 gene displayed an unusual co‐occurrence of five SNPs, as described above, we also examined the geographic substitution of the Quintuple substitution minor allele. Because the five contributing substitutions were found in mere six accessions of our experimentally characterized collection, we analyzed the whole set of genotypes from the Ensembl database (Yates et al. 2022). Populations carrying the quintuple SNP allele often originated from mountainous regions, and the average altitude of their sites of origin was significantly higher than for the reference variant (Figure 7B). Correspondingly, a significant difference was found in multiple climatic variables from the standard BioClim set, especially in parameters reflecting typical features of high altitude biotops, such as increased temperature seasonality, greater difference among temperature and precipitation extremes, and increased solar radiation (Supporting Information S1: Figure S1). The presence of the quintuple substitution allele across three continents is consistent with its selective advantage under high altitude conditions.

### 2.5 Overrepresentation of Some Gene Families Among GWAS Candidates Suggests New Gene Functions

We next examined the sets of loci associated with the four trait groups (trichome shape, trichome density, wall‐related traits and metal‐related traits) for Gene Ontology term association. No enrichment or depletion of any GO categories was found on the *p* = 0.01 significance level, while on the *p* = 0.05 level, a 2.73x enrichment of the Transmembrane signal receptor protein class was found among shape‐associated loci, and a 12.22x enrichment of the snoRNA‐binding molecular function category was observed among metal trait‐associated loci. The biological significance of the latter, however, remains unclear in Light of the apparent low reliability of metal‐related candidate detection (see above).

While inspecting our candidate gene Lists, we noticed the presence of multiple members of some gene families, including those encoding evolutionarily conserved domains of unknown function (DUF). We thus examined several such multigene families for possible enrichment among candidates for individual trait groups. Our enrichment analyses included all seven DUF families represented by two or more genes in at least one trait group, as well as five large gene families containing multiple candidates associated with at least one trait group, namely the F‐box proteins (568 paralogs in Arabidopsis), receptor‐Like protein kinases (307 paralogs), cytochrome P450 isoforms (256 paralogs), cysteine/histidine‐rich C1 domain proteins (153 paralogs) and formins (FH2 proteins, 21 paralogs).

**Figure 8:**
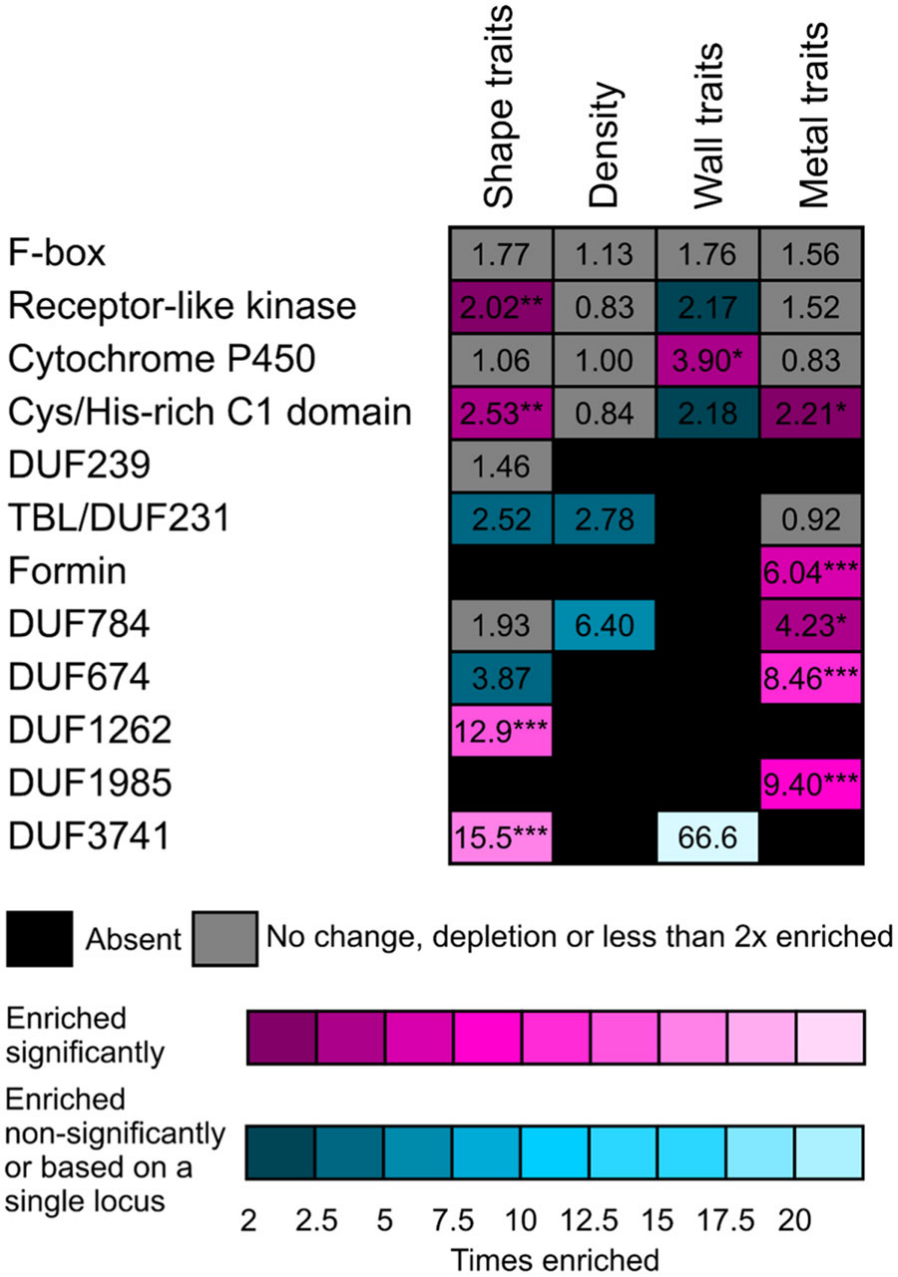
Summary of gene family enrichment analysis for selected gene families. The heatmap shows results of enrichment analysis for selected large gene families and all DUF (domain of unknown function) families that were represented by at least two candidates associated with at least one trait group. Numbers correspond to linear fold enrichment compared with the proportion of the family in question among all Arabidopsis loci (Araport11 genome annotation). Asterisks denote significant deviation from the proportion of the family in the whole genome (determined only for enrichment values of at least two using the *χ* ^2^ test with Benjamini–Hochberg correction for multiplicity; *** *p* ≤ 0.001; ** 0.001 < *p* ≤ 0.01; * 0.01 < *p* ≤ 0.05).

For 9 out of these 12 gene families, we found in at least one category of GWAS candidates a reliable, significant at least 2× enrichment compared to whole genome abundance (Figure 8; Supporting Information S2: Table 9). These include receptor‐Like kinases (minor but significant enrichment in shape traits association), cytochrome P450 (enrichment in cell wall fluorescence‐related traits, compare also Table 4), cysteine/histidine‐rich C1 domain proteins (enrichment in shape and metal accumulation‐related traits), formins, DUF674, DUF784 and DUF1985 proteins (enrichment in metal accumulation traits), as well as the DUF1261 and DUF3741 families that were found in association with trichome shape traits.

**Figure 9:**
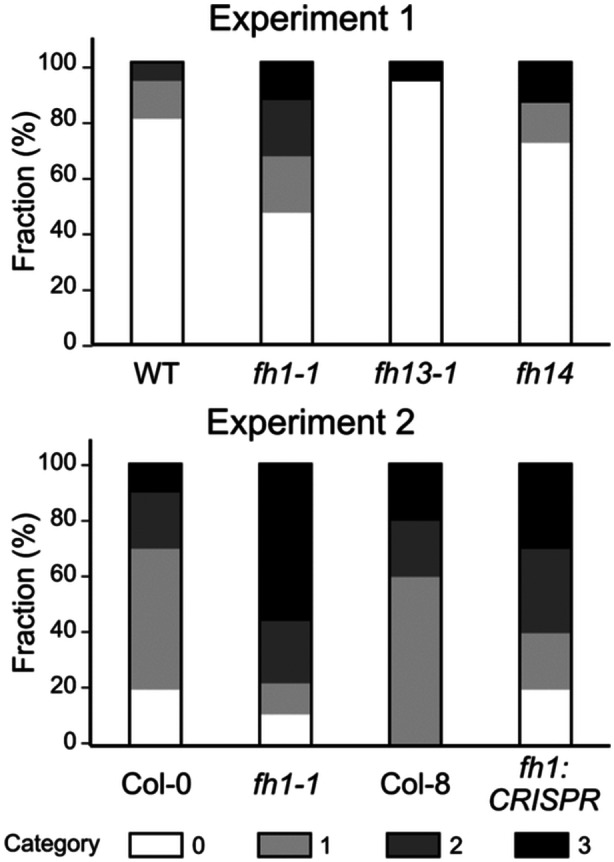
Stomatal metal deposition patterns in selected formin mutants and wild type plants. Data from two independent experiments, involving plants grown at different times under imperfect temperature and humidity control, are shown, each of them based on single‐bLinded evaluation of 9–15 plants per genotype for metal deposition patterns, categorized as in the original GWAS screen. The wild type plants from experiment 1 were WT segregants from the Col‐0 derived T‐DNA mutant population that was the source of our *fh1‐1*, *fh13‐1* and *fh14* lines, while in the second experiment, *fh1‐1* was compared to Col‐0 and the *fh1:CRISPR* mutant to its progenitor genotype Col‐8. The between‐phenotype differences of interest were not statistically significant (Fisher‐Freeman‐Halton test *p* > 0.1).

Making use of available previously characterized loss of function mutants affecting all three formin genes associating with the stomatal metal deposition, that is, *FH1*, *FH13* and *FH14*, we performed single‐blinded evaluation of epidermal metal deposition in these mutants and corresponding wild type plants. The results suggest a tendency towards increased stomatal metal accumulation especially in the *fh1‐1* T‐DNA insertion mutant of the formin gene *FH1*, and possibly also in the CRISPR‐generated loss of function allele of the same gene (Figure 9). Although none of the observed between‐genotype quantitative differences was statistically significant, this observation is consistent with a possible contribution of formins to epidermal metal deposition that would deserve further attention. It also suggests that also the other observed gene family enrichments may be relevant.

### 2.6 Associations Among Candidate Genes Indicate Possible Functional Modules

To uncover functional relationships among genes associating with the studied traits, we scanned the candidate Lists for mutual links recorded in the STRING database (Szklarczyk et al. 2023) that registers both experimentally documented and predicted interactions among genes, such as transcriptional co‐regulation, clustering on the chromosomes, epistasis, or proteins, such as mutual binding.

**Figure 10:**
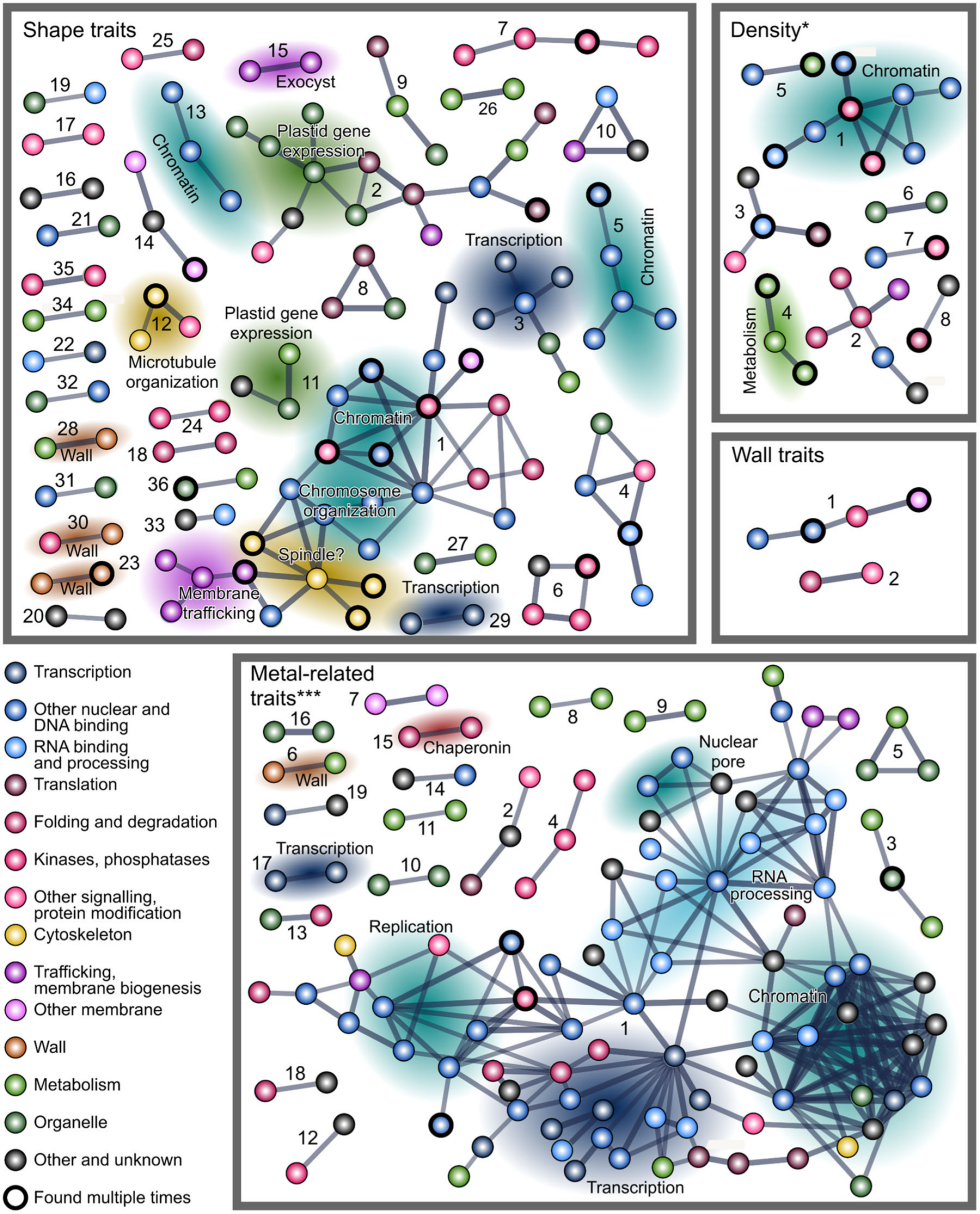
A schematic map of predicted interactions among candidate genes for individual trait groups. Thickness of graph edges reflects strength of evidence. Numbers correspond to the numbering of gene clusters in Supporting Information S2: Table 10. Asterisks denote significant enrichment of interactions compared to a random gene selection, as determined by the STRING algorithm (*** *p* ≤ 0.001; * 0.01 < *p* ≤ 0.05).

Groups of associated genes were found for all four trait groups (Figures 10 and Supporting Information S1: Figure 5, Supporting Information S2: Table 10), but only for trichome density and metal accumulation the numbers of interactions were significantly greater than predicted for a random gene selection (Supporting Information S2: Table 10). Inspection of the found clusters, however, reveals biologically meaningful associations also among candidates Linked to the remaining trait groups. For example, among the shape‐associated genes, cluster 15 consists of Exocyst complex subunits. For density and metal deposition, the clusters notably included mainly genes participating in nuclear functions such as chromatin organization, gene expression, RNA processing or nuclear transport.

**Figure 11:**
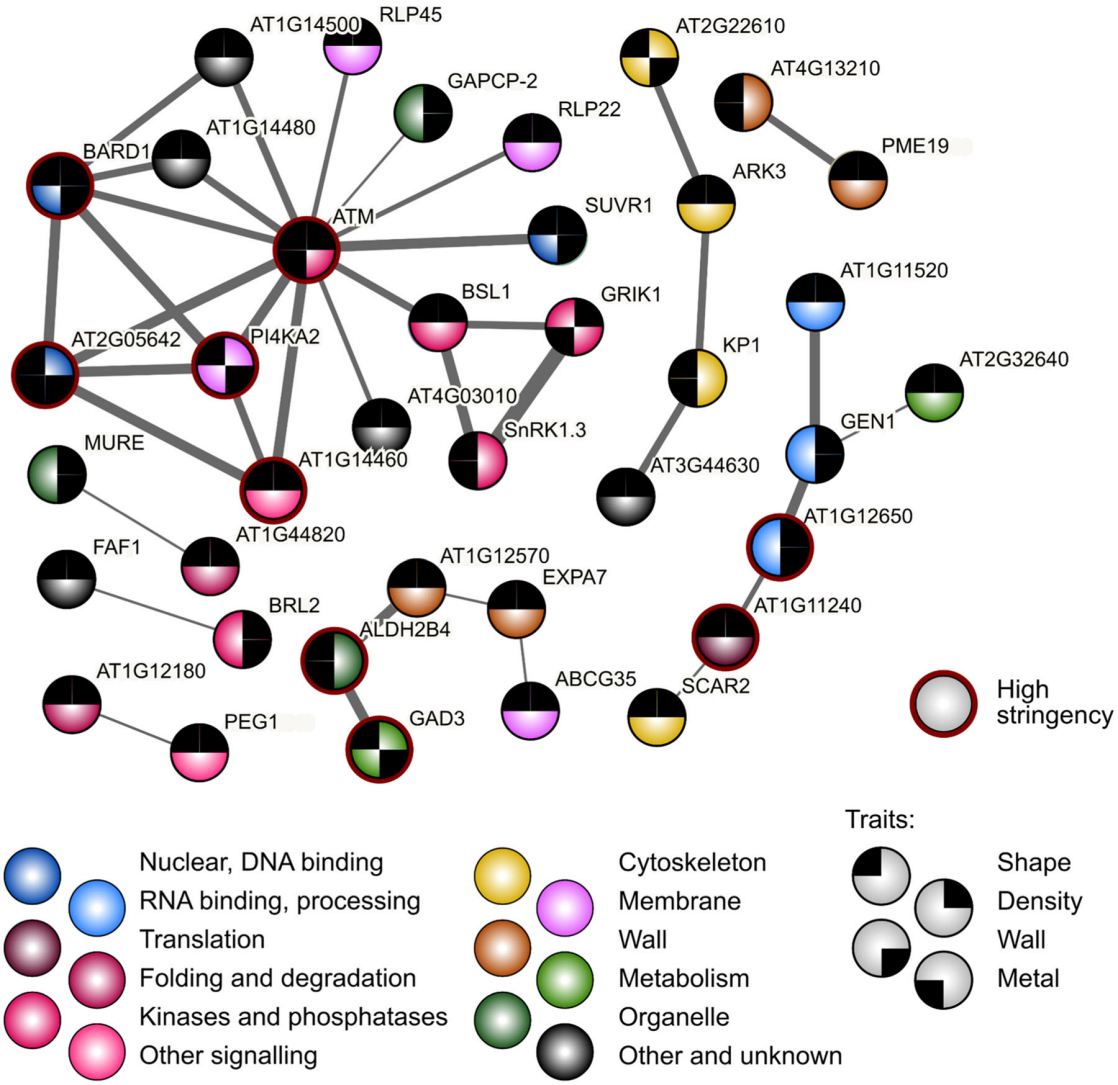
Predicted interactions among candidate genes linked to two or more trait groups. Black segments at graph nodes indicate the trait groups the gene was associated with. Note that the interactions were detected using lower stringency settings than those shown in Figure 9, but a part of the network was found also at high stringency settings (denoted by the red outline of participating nodes). Thickness of graph edges reflects strength of evidence.

Since we noticed that some genes are shared by clusters found for two or more trait groups, we next performed an analogous search for inter‐gene associations among candidates associating with multiple phenotypic trait groups. With somewhat less stringent criteria than those used in the initial interaction searches, this group of genes also exhibited significantly more mutual associations than a random gene selection (Figure 11; Supporting Information S2: Table 10), with one large and two smaller clusters comprising mostly genes associated with shape traits, as well as a small cluster consisting of genes linked to trichome density. We believe that these findings may provide a starting point for exploration of novel regulatory pathways engaged in Arabidopsis epidermal differentiation.

## 3 Discussion

We employed the GWAS approach to explore the genetic basis of *A. thaliana* phenotypic variability, focusing on epidermal traits including trichome development, secondary cell wall composition, production of autofluorescent substances, including those deposited in the cell wall, and metal accumulation. To our knowledge, no similar study has been conducted in Arabidopsis so far, except of a recent report (Arteaga et al. 2022) focusing on trichome patterning and trichome density (the latter also included in our study).

We studied 18 quantitative, semiquantitative or qualitative epidermal traits from 310 predominantly Scandinavian *A. thaliana* accessions from the 1001 Genomes collection (Alonso‐Blanco et al. 2016). These traits can be divided into four groups (trichome shape, trichome density, cell wall composition and metal deposition‐related). All quantitative parameters, as well as most semiquantitative categorical ones, exhibited continuous variability. As expected, traits reflecting trichome size, as well as those describing trichome shape complexity, were mutually positively correlated. Some of the analyzed traits exhibited also significant, albeit usually weak, correlation with selected climatic variables of the sites of origin of our plant accessions. Notably, epidermis autofluorescence was consistently weakly positively correlated with variables reflecting ambient temperature and amount of precipitation. Autofluorescence of plant tissues is largely due to phenolic compounds that might contribute to the defense of plants against microbial pathogens that thrive in warm, wet conditions (Donaldson 2020; Bhattacharya, Sood, and Citovsky 2010). However, the only observed correlation reaching at least the moderate range (*sensu* Schober, Boer, and Schwarte 2018) was a negative one between maximum temperature of the warmest week and trichome stem length, where a biological interpretation was not immediately obvious.

Surprisingly, we noticed strong dithizone staining, presumably detecting zinc and other heavy metal ion accumulation (see, e.g., Frederickson 2003; Srivastava et al. 2014; Peco et al. 2020), in stomatal guard cells of some accessions (but not in the common Col‐0 Line). A similar pattern was reported for Cd accumulation in cadmium‐stressed Col‐0 plants (Zeng et al. 2017). In metazoan cells and in yeast, zinc accumulation is associated with increased secretory activity (Yuan 2011), and this also may be the case in the guard cells, which exhibit vigorous membrane turnover.

The outcome of GWAS studies always presents a compromise between specificity and sensitivity (see e.g. Brachi, Morris, and Borevitz 2011). We found statistically significant missense SNPs associated with phenotypic variability for 10 out of the 18 parameters examined, affecting over 5% of all *A. thaliana* protein‐coding genes. Moreover, none of our candidate genes corresponded to any of the 15 trichome density‐associated loci found in the recent GWAS report (Arteaga et al. 2022), which was based on phenotyping 191 predominantly Iberian *A. thaliana* accessions. This raises concerns whether our screen was specific and sensitive enough to be informative. However, genotype collection size in both studies was obviously far below the saturation threshold. For the similarly sized human genome, GWAS saturation was achieved only with population size several orders larger (Yengo et al. 2022). Our List of trichome density‐associated genes also did not contain any previously characterized trichome patterning genes from the list compiled by Arteaga et al. (2022), nor did we observe significant enrichment of genes encoding most abundant trichome transcripts (Jakoby et al. 2008) or proteins (Huebbers et al. 2022). In case of the metal accumulation traits, where most candidates reflected the guard cell staining, there was no enrichment of the most abundant guard cell transcripts (Leonhardt et al. 2004), or proteins (Zhao et al. 2008). It is not uncommon that candidate genes from GWAS studies are not enriched in, or even are depleted of, known pathway genes. This would be consistent with the assumption that mutations in large effects genes, which typically tend to be the known genes, might not be beneficial in natural environments (Ristova et al. 2018). Taken together, we beLieve that at least some of our candidates are biologically relevant, because the candidate genes set, both as a whole and for each trait group except metal accumulation, was significantly (or, in one case marginally significantly) depleted of transposon‐borne loci, whose SNPs, unLike polymorphisms concerning transposon presence or position (see, e.g., Yan et al. 2022), are rather unlikely to have phenotypic effects. Thus, the sets of trichome shape, density and probably also wall trait‐associated GWAS candidates are Likely to contain biologically meaningful genes, while the relevance of candidates found on the basis of metal‐related traits remains questionable.

We recovered five loci with well documented mutant phenotypes consistent with the associations identified in our study. First of them is the AT2G20190/CLASP gene coding for a microtubule‐associated protein whose loss reduces trichome branch number (Pietra et al. 2013), In this gene, a SNP causing a conserved M1399L substitution in the C‐terminal Armadillo‐like repeat correlated with longer trichome stems. This substitution occurred more frequently in accessions from locations with higher Bio05 climate variable, reflecting the maximum environmental temperature at the site of the accession's origin. CLASP stabilizes microtubules and consequently affects cell division and cell expansion (Ambrose et al. 2007; Pietra et al. 2013). The stability of Arabidopsis microtubule assemblies can be impaired by high temperatures, resulting in defective meiosis (De Storme and Geelen 2020). Although a direct meiotic role of CLASP has not been documented, it is conceivable that the observed environmentally correlated allele distribution may reflect selection on the basis of a phenotype unrelated to epidermal development. This may be the case also for other loci with environmental bias in allele distribution, such as SYT1, which codes for a synaptotagmin involved in repair of membrane breaks caused by freezing (Yamazaki et al. 2008) and in defense against pathogens (Levy, Zheng, and Lazarowitz 2015; Kim et al. 2016) whose abundance could be linked to environmental conditions.

The second candidate gene with a relevant mutant phenotype is AT2G38440/DIS3, encoding a subunit of the WAVE complex regulating Arp2/3‐mediated actin nucleation. Its mutation causes a characteristic distorted trichome shape (Basu et al. 2005). Here, a conserved I1265V substitution in an area immediately preceding the WASP homology motif associated with reduced trichome circularity and solidity, as well as lower trichome density. The third gene, AT5G49830/EXO84B, codes for an exocyst complex subunit with a pleiotropic mutant phenotype that includes trichome deformation (Kulich et al. 2015; Fendrych et al. 2010). A non‐conservative E123G substitution at a variable sequence position of this locus (see Cvrčková et al. 2012) associated with decreased trichome circularity and soLidity. A non‐conservative S93N substitution in a variable segment of another exocyst subunit, AT3G09520/EXO70H4, involved in metal deposition in the trichome cell wall (Kulich et al. 2015), associated with increased guard cell metal staining.

Last but not least, we found two conservative substitutions (S626A and D671E) associating with a decreae of trichome autofluorescence and also trichome density in AT3G28430/GFS9/TT9, which codes for a Golgi‐associated peripheral membrane protein involved in membrane trafficking, vacuole development and flavonoid accumulation whose mutation results in altered autofluorescence (Ichino et al. 2014; Ichino et al. 2020). These mutations are frequently co‐occurring with additional three substitutions, constituting a quintuple SNP minor allele, which appears to be present in multiple accessions of mountain origin across the globe. It is tempting to speculate that a variant with reduced cell wall autofluorescence may have altered flavonoid distribution among cell compartments, and perhaps enhanced vacuolar accumulation of anthocyanins that might bring a selective advantage under high altitude conditions characterized by increased UV stress, resulting in the fixation of this minor allele in mountain habitats. The presence of five identical SNPs in geographically disparate populations points towards this allele's unique origin and subsequent elimination in most populations, rather than parallel independent mutations analogous to those found for example in naturally occurring “green revolution dwarf” Arabidopsis mutants (Barboza et al. 2013). The quintuple substitution allele of GFS9/TT9 would clearly deserve a follow‐up experimental study.

Among the loci associated with trichome shape‐related traits and also highly expressed in trichomes were found three genes participating in the biosynthesis of cutin and/or cuticular waxes. AT1G01600/CYP86A4 encodes a cytochrome P450 paralog, associates also with the trichome autofluorescence trait. Its expression is controlled by a transcription network that regulates also trichome branching (Camoirano et al. 2020; see also Berhin, Nawrath, and Hachez 2022). Remarkably, the cytochrome P450 gene family is significantly over‐represented among cell wall trait‐associated candidates, also consistent with its role in cell wall biogenesis. The second trichome shape‐related candidate with high trichome expression, AT1G12570, encodes a glucose‐methanol‐choLine oxidoreductase related to the maize IPE1 gene participating in pollen exine development, which is co‐regulated with CYP86A4 (Kannangara et al. 2007). Polymorphisms in both loci correlate also with variation in trichome density. Remarkably, trichome patterning is altered in some cuticle biogenesis mutants (Berhin, Nawrath, and Hachez 2022), suggesting cuticle contribution to the mechanical cell coupLing and tissue patterning (Reynoud et al. 2021). The third locus implicated in cuticle biogenesis is AT1G76690/OPR2, a 12‐oxophytodienoic acid reductase co‐regulated with the previous two loci (Suh et al. 2005), which only associated with variation of trichome stem length.

Relevant candidates were also found among guard cell‐expressed genes. The loci associated with increased guard cell metal accumulation and highly expressed in guard cells include AT3G13772/TMN7, encoding a membrane protein whose heterologous expression causes yeast to accumulate copper (Hegelund et al. 2010), and AT5G39040/ALS1, coding an ABC transporter whose loss leads to increased aluminum sensitivity (Larsen et al. 2007). Another gene impLicated in stomatal metal accumulation, AT3G13460/ECT2, encodes a mRNA stability‐controlLing protein whose mutational loss affects trichome morphogenesis (Scutenaire et al. 2018; Wei et al. 2018). Identification of this locus, which is intensively expressed in guard cells (Zhao et al. 2008) in the context of a guard cell‐related phenotype indicates its broader role in epidermal development.

Eighty further candidates with Likely roles in biologically relevant processes such as membrane trafficking, cytoskeletal organization or cell wall biogenesis were recovered. Among them are several members of the trichome birefringence‐like (TBL) gene family whose founding member was discovered due to altered trichome cell wall optical properties (Bischoff et al. 2010), an additional exocyst subunit, multiple myosin‐ or kinesin‐related proteins, several extensins and expansins, and about 20 cell wall modifying enzymes.

While these observations can be considered largely confirmatory, our candidate gene Lists are significantly enriched in members from several multigene families not yet reported to participate in relevant aspects of epidermal development. The cysteine/histidine‐rich C1 domain proteins, a group of nucleic acid binding proteins with a possible role in plant defense (Hwang et al. 2014) were enriched among trichome shape and metal accumulation‐Linked candidates, and receptor‐Like kinases (RLKs) associated with trichome shape traits, although no overlaps with a published list of trichome‐expressed LRR‐RLKs (Wu et al. 2016), representing one of the RLK family branches, were detected. Formins, evolutionarily conserved cytoskeletal organizers that also engage in endomembrane dynamics (see Cvrčková, Ghosh, and Kočová 2024), surprisingly associated with stomatal metal accumulation, including the main housekeeping Class I formin FH1/AT3G25500 (Rosero et al. 2016; Oulehlová et al. 2019; Cifrová et al. 2020). Two candidate conservative amino acid substitutions were found in FH1 (L5F within the membrane insertion signal and D502E in a variable cytoplasmic segment). Observations in two loss of function *fh1* mutants suggest a trend towards increased metal accumulation in guard cells. Last but not least, members of five hitherto uncharacterized ‘domain of unknown function’ (DUF) famiLies associated with either trichome shape or metal accumulation traits, including the prolamin‐related DUF784 (Zhang 2009), suggesting their participation in relevant developmental pathways.

Although our Gene Ontology term enrichment analyses did not indicate links between the candidates and specific cellular functions, we observed a significant increase in mutual genetic, physical and functional interactions recorded in the STRING database (Szklarczyk et al. 2023) among candidates for the trichome density and metal deposition traits, as well as for candidates associated with multiple traits, compared to a random gene sample. Remarkably, the interacting gene clusters consisted predominantly of genes participating in nuclear functions. A prominent cluster of candidates associated with two or more trait groups comprising mainly signalling and regulatory proteins was centred on a homolog of the mammalian ATM kinase engaged in DNA damage response (Lee and Paull 2021). In plants, ATM homologs participate in DNA repair and UV stress response (Shi and Liu 2021), but also in reaction to exogenous DNA (Vega‐Muñoz et al. 2023). Among genes associated with more than one trait category, we also found smaller clusters of shape‐associated genes presumably engaged in cytoskeletal and cell wall‐related functions, as well as an additional cluster comprising several nuclear proteins and containing genes linked to trichome density.

In summary, our screen identifies several candidate gene families and functional gene clusters that would deserve experimental study to verify their possible roles in epidermal development, possibly leading to uncovering new genetic players in cell morphogenesis and cell differentiation, but also in functions possibly relevant for environmental adaptation under natural conditions.

## 4 Materials and Methods

### 4.1 Plant Material

A set of 310 natural *Arabidopsis thaliana* accessions from the 1001 Genomes collection (Alonso‐Blanco et al. 2016), has been included in our study (Supporting Information S2: Table 1). Predominantly, genotypes of North‐European origin were chosen to reduce the effect of geographical differences in population structure and related parameters reducing GWAS efficacy (see Gloss et al. 2022). Climate variables data for the locations of origin vere retrieved in the form of standardized BioClim variables from the CliMond database (Kriticos et al. 2012).

Seeds were steriLized with chlorine gas, stratified in tubes at 4°C for 3 days in the dark and sown into standard soil substrate pre‐treated with Confidor to prevent blackfly infestation. Plants were grown in growth chambers under controlled long‐day conditions (16 h light, 23°C/8 h darkness, 18°C; relative air humidity 60%). Three seedlings per genotype were planted into individual pots at the first true leaf stage. Leaves were collected at the age of 5 weeks (at which point plants of some genotypes were beginning to bolt).

For mutant genotype verification for selected formin‐encoding genes, homozygous plant lines carrying previously characterized loss of function mutants *fh1‐1* (SALK‐032981; Rosero, Žárský, and Cvrčková 2013), *fh13‐1* (SALK_064291C; Kollárová, Baquero Forero, and Cvrčková 2021), and *fh14‐1* (SALK_058886; Li et al. 2010), available from NASC (RRID: SCR_004576), as well as *fh1:CRISPR* (generated in our laboratory (see Cifrová et al. 2020), with corresponding congenic wild type plants were used.

### 4.2 Leaf Sample Processing

Three approximately fingernail‐sized mature, non‐senescent rosette leaves per plant (i.e., nine leaves per genotype) were harvested, resulting in three pooled samples, each containing one leaf from each individual. Samples were stored for at least 2 days before further processing or observation.

For trichome shape evaluation and for determination of callose content in trichomes, leaves were harvested into a tube containing 1x phosphate‐buffered saline (PBS) and 100 mM EGTA. Trichomes were isolated and stained for callose by aniline blue as described previously (Kulich et al. 2015). In genotypes where trichomes failed to detach from the leaves (further referred to as ‘shaveproof’), whole leaves were stained for callose using an analogous procedure (Kulich et al. 2018). This in situ staining was also used for additional documentation, such as the photos shown in Figure 1.

For visualization of autofluorescence and for determination of additional morphological parameters, leaves were pressed adaxial side up between two microscope slides and left to dry up to induce breakage of trichomes resulting in increase of autofluorescence (Kulich et al. 2018).

For visualization of metal content, leaves were collected into a tube containing 3 mL of acetone and stored for at least several days. On the day of observation, they were stained by addition of diphenylthiocarbazone (dithizone) reagent freshly prepared from 1.5 mg of dithizone, 1 mL of distilled water and one to two drops of glacial acetic acid (Seregin and Ivanov 1997). After at least 1 h but not more than 6 h of staining, leaves were briefly washed in distilled water and mounted in water on microscopy slides for observation.

### 4.3 Microscopy and Imaging

Microscopic images were acquired using a Nikon Eclipse 90i fluorescence microscope equipped with PlanApo 4x/0.2 objective and Nikon DsFi 2 camera as described previously (Kulich et al. 2018). For autofluorescence and callose fluorescence detection, UV‐B excitation was employed; in case of callose staining, an additional image was acquired in polarized light for trichome visualization. Leaves stained for metal detection were imaged using bright field settings.

If whole leaves were photographed, tilling images covering at least a half of the leaf lengthwise were automatically stitched from multiple frames, which were generated as maximum projections of three images with 50 μm of Z distance, using the Nikon Imaging Software (NIS Elements AR). Additional image processing was performed using the Fiji platform (Schindelin et al. 2012).

### 4.4 Epidermal Phenotypes Determination

An overview of phenotypic trauts analyzed in our screen is provided in Table 1.

The first group of parameters was determined from photos of callose stained trichomes. At least 25 interactively selected clearly visible trichomes from three leaves of three plants (detached, or, in shaveproof genotypes, 8–9 in situ trichomes per leaf, selected across its diagonal) were evaluated. Trichome shape parameters (area, circularity, soLidity, length and perimeter) were determined from binary images generated by thresholding polarized light photos using the Yen method as implemented in the Fiji software; the procedure was partially automated using recorded macros to increase throughput, with individual trichome selection being the only manual step. Trichome length was measured manually as the longest trichome dimension. Average parameter values from at least 25 trichomes were recorded for these parameters. Callose content was measured as the fraction of trichome area stained for callose, determined using built‐in functions of Fiji from binary images generated by thresholding fluorescence and polarized light photos using the Yen method, again with the aid of recorded macros and manual selection of individual trichomes. The reported value for each accession is the sum of percentage values from 25 trichomes.

The second group of parameters was determined from fluorescence microscopy images of dried whole leaves, taken in the autofluorescence channel. Trichome stem length was measured using the NIS elements software with manual endpoint identification; average values from at least 25 clearly visible trichomes from 3 leaves, selected 7–9 per leaf across its diagonal, are reported. Additional parameters (trichome autofluorescence, overall leaf epidermis autofluorescence, autofluorescence colour, trichome density and typical number of trichome branches) were visually categorized (see Table 1).

A third group of parameters reflects visually detectable patterns of metal accumulation, categorized as described in Table 1. This group includes the presence of a metal‐enriched Ortmannian ring at the bottom part of the trichome stem (Kulich et al. 2015), presence of a visible metal staining gradient in the trichome stem, presence of metal staining at the trichome base, diffuse epidermal staining outside trichomes and staining of stomatal guard cells.

The final categorical parameter, denoted as ‘shaveproof’, reflects the resistance of trichomes against mechanical detachment during staining for callose.

All categorical values were based on the typical appearance of the epidermis of three leaves. Glabrous leaves were attributed the value ‘0’ for all categorical variables. In theexceptional cases where noticeable differences were seen among the three leaves of a given accession, the majority phenotype was recorded.

Phenotype data for a subset of accessions and traits have been independently rescreened by different team members for the purpose of broad sense heritability estimation (Supporting Information S2: Table 2); this also documented good inter‐observer replicability of phenotype determination (Supporting Information S1: Figure 6).

### 4.5 Phenotypic Data Processing and Initial Analyses

Primary phenotypic data were assembled into a structured spreadsheet and deposited in the pubLic AraPheno database (Togninalli et al. 2020) as Study No. 126 (Bezvoda 2023). Broad sense heritabiLity (H2 = VG/VP), that is, the proportion of phenotypic variation (VP) due to genetic variation (VG) estimated from the between‐ and within‐Line phenotypic variance, was calculated from trait values of all individuals in this data set (Alonso‐Díaz et al. 2021).

Statistical analysis of phenotypic data and phenotype/environmental parameter correlation analyses were carried out by the Pandas data analysis toolbox (Reback et al. 2021) and pair plots of scatterplots were created by the Seaborn visualization tool (Waskom 2021) v. 0.11.2. Correlation strength is reported using criteria from Schober, Boer and Schwarte (2018).

Principal component analysis (PCA) has been performed using the PAST software (Hammer, Harper, and Ryan 2001) v. 4.11 using the correlation matrix method. All quantitative or semi‐quantitative parameters were treated as ordinal variables, the qualitative ‘autofluorescence color’ and ‘shaveproof’ traits were handled as nominal.

### 4.6 GWAS Analyses and Genomic Data Processing

Phenotype values for each trait were uploaded to the online GWAS application (Seren et al. 2012; Seren 2015) and correlation analyses were run at 5% FDR threshold with all possible combinations of input data transformations and statistical methods available with default settings except the minor allele count (MAC) value that has been lowered to 5. The 1001 Full sequence Data set (TAIR v. 9) was used as the source of genotype information. After initial manual exploration of resulting Manhattan plots, the process of analyzing data was automated.

Raw result data files were downloaded manually and used for subsequent steps. All SNPs identified as significant by at least one method were considered. Additional information about each significant hit was acquired from the GWAS application by web scraping using the Python programming language v. 3.8 (Python Software Foundation 2024) and driver for Google Chrome web browser (Google 2024). Locus annotation was based on TAIR9.0 to maintain compatibility with the 1001 genomes data set. All intergenic hits were discarded from subsequent steps of data processing. Hits were categorized according to the position of the SNP in corresponding gene (INTRON, NON_SYNONYMOUS_CODING, START_LOST, STOP_GAINED, STOP_LOST, SYNONYMOUS_CODING, SYNONYMOUS_STOP, UTR_3_PRIME, UTR_5_PRIME).

Annotation of loci that were subjected to further investigation was individually updated by manual searches of the Araport 11 genome annotation (Cheng et al. 2017) accessed via the BAR Thalemine portal (Pasha et al. 2020).

To determine the direction of phenotypic parameter cHanges for individual minor allele amino acid substitutions, lists of accessions carrying specific substitutions retrieved from the Ensembl resource (Yates et al. 2022) were used to identify subsets of our accessions carrying individual sequence variants. Average values of the phenotypic parameters of question were subsequently determined for each such variant.

### 4.7 Geographic Mapping of Allele Distribution

Lists of accessions carrying specific alleles of selected loci for the purpose of environmentally correlated allele distribution analyses and for map generation were generated from Ensembl data as described above. Geographic coordinate‐based maps were created using Python Folium (Python Visualisation 2024) or Google Maps tools.

### 4.8 Enrichment and Depletion Analyses of GWAS Candidate Gene Lists

To determine whether lists of candidate genes identified in the GWAS are enriched or depleted for specific gene groups, we used the Arabidopsis genome (Araport 11 version) and compared our data lists after manual annotation with the following gene lists.

As mobile element‐derived genes, those with annotations containing the word ‘transposable’ were considered.

As genes with high transcript levels in trichomes, we considered the 164 genes listed as encoding 5% most abundant transcripts in the mature trichome transcriptome (Jakoby et al. 2008).

As genes with guard cell‐specific transcription patterns, we considered the list of loci specifically upregulated in the guard cells compared to the mesophyll (Leonhardt et al. 2004).

As genes encoding trichome‐abundant proteins, we selected genes from the recently published Arabidopsis trichome proteome study (Huebbers et al. 2022) using the following criteria. A protein had to be significantly enriched in at least two of the four reported proteomic experiments, at least in one case by a factor of two and more, while it was not depleted in any experiment. These criteria produced a list of 455 loci.

For genes encoding guard cell‐abundant proteins, we considered the list from a published guard cell proteome study (Zhao et al. 2008).

For gene family enrichment, we included all gene family members as identified by keyword search of candidate gene annotations. Family member counts were based either on Literature, as in the case of F‐box proteins (Kuroda et al. 2002), on TAIR (Reiser et al. 2024) Gene FamiLies annotations (as in the case of receptor‐like protein kinases and FH2 proteins), or estimated by keyword searches of the Araport 11 annotations (for all DUFs).

Genes from each list were identified among the GWAS candidates and significance of any observed enrichment or depletion was evaluated using pairwise *χ* ^2^ test (Stagroom 2024) with Benjamini–Hochberg correction for multipLicity performed using an online calculator (Radua, Albajes‐Elsagirre, and Fortea 2024).

### 4.9 Verification of Candidates

Loss‐of‐function mutants in candidate genes were grown alongside corresponding wild type plants and leaf samples were harvested, processed and imaged as described above for the main ecotype screen. To minimize effects of observer's expectation bias, a single‐bLinded experimental design was employed, with one member of the team performing the imaging and others quantitatively evaluating microphotographs with coded labels without knowledge of the plant's genotype. After decoding the identity of the samples, significance of between‐genotype differences in the relevant categorical parameters was estimated by the *χ* ^2^ test (or Fisher's exact test in cases where some categories exhibited zero counts) using online calculators (Stagroom 2024; Vasavada 2016).

### 4.10 Mapping of Protein–Protein Interactions Among Candidature Gene Products

To gain initial insight into the interactions among candidate genes, the full network STRING protein–protein interaction database v. 11.5 (Szklarczyk et al. 2023) was searched with non‐redundant lists of genes associated with the indicated trait groups as queries, using high confidence (score = 0.7) and high stringency (FDR = 1%) settings. Statistical significance of interaction enrichment was obtained during this search. Resulting interaction networks were exported both as tables (further processed in Excel to generate cluster and node lists that were subsequently annotated as described above) and as vector images that were manually edited (to remove unlinked nodes and tidy up the layout), annotated and coloured.

Mapping of interactions among the subset of candidates associated with multiple trait groups was performed analogously except that the confidence threshold was lowered to medium (score = 0.4), that is, to the default settings of the STRING database search tool.

## Conflicts of Interest

The authors declare no conflicts of interest.

## Supporting information

Supporting information.

Supporting information.

## Data Availability

Primary data from the phenotype screen are available in the AraPheno database (https://doi.org/10.21958/study:126). Additional data supporting the findings of this study are available in the Supporting Information of this article.

## References

[pce15357-bib-0001] Alonso‐Blanco, C. , J. Andrade , C. Becker , et al. 2016. “1,135 Genomes Reveal the Global Pattern of Polymorphism in *Arabidopsis thaliana* .” Cell 166, no. 2: 481–491. 10.1016/j.cell.2016.05.063. 27293186 PMC4949382

[pce15357-bib-0002] Alonso‐Díaz, A. , S. B. Satbhai , R. de Pedro‐Jové , et al. 2021. “A Genome‐Wide Association Study Reveals Cytokinin as a Major Component in the Root Defense Responses Against *Ralstonia solanacearum* .” Journal of Experimental Botany 72, no. 7: 2727–2740. 10.1093/jxb/eraa610. 33475698 PMC8006551

[pce15357-bib-0003] Ambrose, J. C. , T. Shoji , A. M. Kotzer , J. A. Pighin , and G. O. Wasteneys . 2007. “The Arabidopsis Clasp Gene Encodes a Microtubule‐Associated Protein Involved in Cell Expansion and Division.” Plant Cell 19, no. 9: 2763–2775. 10.1105/tpc.107.053777. 17873093 PMC2048705

[pce15357-bib-0004] Arteaga, N. , B. Méndez‐Vigo , A. Fuster‐Pons , et al. 2022. “Differential Environmental and Genomic Architectures Shape the Natural Diversity for Trichome Patterning and Morphology in Different Arabidopsis Organs.” Plant, Cell & Environment 45, no. 10: 3018–3035. 10.1111/pce.14308. PMC9541492 35289421

[pce15357-bib-0005] Barboza, L. , S. Effgen , C. Alonso‐Blanco , et al. 2013. “Arabidopsis Semidwarfs Evolved From Independent Mutations in GA20ox1, Ortholog to Green Revolution Dwarf Alleles in Rice and Barley.” Proceedings of the National Academy of Sciences 110, no. 39: 15818–15823. 10.1073/pnas.1314979110. PMC3785751 24023067

[pce15357-bib-0006] Basu, D. , J. Le , S. E. D. El‐Essal , et al. 2005. “DISTORTED3/SCAR2 Is a Putative Arabidopsis Wave Complex Subunit That Activates the Arp2/3 Complex and Is Required for Epidermal Morphogenesis.” Plant Cell 17, no. 2: 502–524. 10.1105/tpc.104.027987. 15659634 PMC548822

[pce15357-bib-0007] Bates, G. W. , D. M. Rosenthal , J. Sun , et al. 2012. “A Comparative Study of the *Arabidopsis thaliana* Guard‐Cell Transcriptome and Its Modulation by Sucrose.” PLoS One 7, no. 11: e49641. 10.1371/journal.pone.0049641. 23185391 PMC3504121

[pce15357-bib-0008] Berhin, A. , C. Nawrath , and C. Hachez . 2022. “Subtle Interplay Between Trichome Development and Cuticle Formation in Plants.” New Phytologist 233, no. 5: 2036–2046. 10.1111/nph.17827. 34704619

[pce15357-bib-0009] Bezvoda, R. 2023. “AraPheno Study: Trichome_development_epidermis_metal_traits.” 10.21958/study:126.

[pce15357-bib-0010] Bhattacharya, A. , P. Sood , and V. Citovsky . 2010. “The Roles of Plant Phenolics in Defence and Communication During Agrobacterium and Rhizobium Infection.” Molecular Plant Pathology 11, no. 5: 705–719. 10.1111/j.1364-3703.2010.00625.x. 20696007 PMC6640454

[pce15357-bib-0011] Bischoff, V. , S. Nita , L. Neumetzler , et al. 2010. “Trichome Birefringence and Its Homolog AT5G01360 Encode Plant‐Specific DUF231 Proteins Required for Cellulose Biosynthesis in Arabidopsis.” Plant Physiology 153, no. 2: 590–602. 10.1104/pp.110.153320. 20388664 PMC2879772

[pce15357-bib-0012] Brachi, B. , G. P. Morris , and J. O. Borevitz . 2011. “Genome‐Wide Association Studies in Plants: The Missing Heritability Is in the Field.” Genome Biology 12, no. 10: 232. 10.1186/gb-2011-12-10-232. 22035733 PMC3333769

[pce15357-bib-0013] Brocker, C. , M. Vasiliou , S. Carpenter , et al. 2013. “Aldehyde Dehydrogenase (ALDH) Superfamily in Plants: Gene Nomenclature and Comparative Genomics.” Planta 237, no. 1: 189–210. 10.1007/s00425-012-1749-0. 23007552 PMC3536936

[pce15357-bib-0014] Camoirano, A. , A. L. Arce , F. D. Ariel , A. L. Alem , D. H. Gonzalez , and I. L. Viola . 2020. “Class I TCP Transcription Factors Regulate Trichome Branching and Cuticle Development in Arabidopsis.” Journal of Experimental Botany 71, no. 18: 5438–5453. 10.1093/jxb/eraa257. 32453824

[pce15357-bib-0015] Cheng, C. Y. , V. Krishnakumar , A. P. Chan , F. Thibaud‐Nissen , S. Schobel , and C. D. Town . 2017. “Araport11: A Complete Reannotation of the *Arabidopsis thaliana* Reference Genome.” Plant Journal 89, no. 4: 789–804. 10.1111/tpj.13415. 27862469

[pce15357-bib-0016] Cifrová, P. , D. Oulehlová , E. Kollárová , et al. 2020. “Division of Labor Between Two Actin Nucleators—The Formin FH1 and the ARP2/3 Complex—in *Arabidopsis* Epidermal Cell Morphogenesis.” Frontiers in Plant Science 11: 148. 10.3389/fpls.2020.00148. 32194585 PMC7061858

[pce15357-bib-0017] Crowell, D. N. , D. H. Huizinga , A. K. Deem , C. Trobaugh , R. Denton , and S. E. Sen . 2007. “ *Arabidopsis thaliana* Plants Possess a Specific Farnesylcysteine Lyase That Is Involved in Detoxification and Recycling of Farnesylcysteine.” Plant Journal 50, no. 5: 839–847. 10.1111/j.1365-313X.2007.03091.x. 17425716

[pce15357-bib-0018] Cvrčková, F. , R. Ghosh , and H. Kočová . 2024. “Transmembrane Formins as Active Cargoes of Membrane Trafficking.” Journal of Experimental Botany 75, no. 12: 3668–3684. 10.1093/jxb/erae078. 38401146 PMC11194305

[pce15357-bib-0019] Cvrčková, F. , M. Grunt , R. Bezvoda , et al. 2012. “Evolution of the Land Plant Exocyst Complexes.” Frontiers in Plant Science 3: 159. 10.3389/fpls.2012.00159. 22826714 PMC3399122

[pce15357-bib-0020] De Storme, N. , and D. Geelen . 2020. “High Temperatures Alter Cross‐Over Distribution and Induce Male Meiotic Restitution in *Arabidopsis thaliana* .” Communications Biology 3, no. 1: 187. 10.1038/s42003-020-0897-1. 32327690 PMC7181631

[pce15357-bib-0021] Dettmer, J. , A. Hong‐Hermesdorf , Y. D. Stierhof , and K. Schumacher . 2006. “Vacuolar H+‐Atpase Activity Is Required for Endocytic and Secretory Trafficking in Arabidopsis.” Plant Cell 18, no. 3: 715–730. 10.1105/tpc.105.037978. 16461582 PMC1383645

[pce15357-bib-0022] Donaldson, L. 2020. “Autofluorescence in Plants.” Molecules 25, no. 10: 2393. 10.3390/molecules25102393. 32455605 PMC7288016

[pce15357-bib-0023] Eggers, R. , A. Jammer , S. Jha , et al. 2021. “The Scope of Flavin‐Dependent Reactions and Processes in the Model Plant *Arabidopsis thaliana* .” Phytochemistry 189: 112822. 10.1016/j.phytochem.2021.112822. 34118767

[pce15357-bib-0024] Fendrych, M. , L. Synek , T. Pečenková , et al. 2010. “The Arabidopsis Exocyst Complex Is Involved in Cytokinesis and Cell Plate Maturation.” Plant Cell 22, no. 9: 3053–3065. 10.1105/tpc.110.074351. 20870962 PMC2965533

[pce15357-bib-0025] Frederickson, C. 2003. “Imaging Zinc: Old and New Tools.” Science's STKE: Signal Transduction Knowledge Environment 2003, no. 182: 18. 10.1126/stke.2003.182.pe18. 12746547

[pce15357-bib-0026] Gao, W. , C. Guo , J. Hu , J. Dong , and L. H. Zhou . 2021. “Mature Trichome Is the Earliest Sequestration Site of Cd Ions in *Arabidopsis thaliana* Leaves.” Heliyon 7, no. 7: e07501. 10.1016/j.heliyon.2021.e07501. 34307941 PMC8287149

[pce15357-bib-0027] Gloss, A. D. , A. Vergnol , T. C. Morton , P. J. Laurin , F. Roux , and J. Bergelson . 2022. “Genome‐Wide Association Mapping Within a Local *Arabidopsis thaliana* Population More Fully Reveals the Genetic Architecture for Defensive Metabolite Diversity.” Philosophical Transactions of the Royal Society of London, Series B: Biological Sciences 377, no. 1855: 20200512. 10.1098/rstb.2020.0512. 35634919 PMC9149790

[pce15357-bib-0028] Google . 2024. “ChromeDriver.” Chrome for Developers. https://chromedriver.chromium.org/.

[pce15357-bib-0029] Guo, C. , J. Hu , W. Gao , et al. 2022. “Mechanosensation Triggers Enhanced Heavy Metal Ion Uptake by Non‐Glandular Trichomes.” Journal of Hazardous Materials 426: 127983. 10.1016/j.jhazmat.2021.127983. 34923380

[pce15357-bib-0030] Hammer, Ø. , D. A. T. Harper , and P. D. Ryan . 2001. “Past: Paleontological Statistics Software Package for Education and Data Dnalysis.” Palaeontologia Electronica 4, no. 1: 4. http://palaeo-electronica.org/2001_1/past/issue1_01.htm.

[pce15357-bib-0031] Han, G. , Y. Li , Z. Yang , C. Wang , Y. Zhang , and B. Wang . 2022. “Molecular Mechanisms of Plant Trichome Development.” Frontiers in Plant Science 13: 910228. 10.3389/fpls.2022.910228. 35720574 PMC9198495

[pce15357-bib-0032] Harada, E. , J. A. Kim , A. J. Meyer , R. Hell , S. Clemens , and Y. E. Choi . 2010. “Expression Profiling of Tobacco Leaf Trichomes Identifies Genes for Biotic and Abiotic Stresses.” Plant & Cell Physiology 51, no. 10: 1627–1637. 10.1093/pcp/pcq118. 20693332

[pce15357-bib-0033] Hauser, M. T. 2014. “Molecular Basis of Natural Variation and Environmental Control of Trichome Patterning.” Frontiers in Plant Science 5: 320. 10.3389/fpls.2014.00320. 25071803 PMC4080826

[pce15357-bib-0034] Hauser, M. T. , B. Harr , and C. Schlötterer . 2001. “Trichome Distribution in *Arabidopsis thaliana* and Its Close Relative *Arabidopsis lyrata*: Molecular Analysis of the Candidate Gene GLABROUS1.” Molecular Biology and Evolution 18, no. 9: 1754–1763. 10.1093/oxfordjournals.molbev.a003963. 11504855

[pce15357-bib-0035] Hegelund, J. N. , T. P. Jahn , L. Baekgaard , M. G. Palmgren , and J. K. Schjoerring . 2010. “Transmembrane Nine Proteins in Yeast and Arabidopsis Affect Cellular Metal Contents Without Changing Vacuolar Morphology.” Physiologia Plantarum 140, no. 4: 355–367. 10.1111/j.1399-3054.2010.01404.x. 20681974

[pce15357-bib-0036] Hilscher, J. , C. Schlötterer , and M. T. Hauser . 2009. “A Single Amino Acid Replacement in ETC2 Shapes Trichome Patterning in Natural Arabidopsis Populations.” Current Biology 19, no. 20: 1747–1751. 10.1016/j.cub.2009.08.057. 19818620 PMC2864576

[pce15357-bib-0037] Horton, M. W. , N. Bodenhausen , K. Beilsmith , et al. 2014. “Genome‐Wide Association Study of *Arabidopsis thaliana* Leaf Microbial Community.” Nature Communications 5: 5320. 10.1038/ncomms6320. PMC4232226 25382143

[pce15357-bib-0038] Huebbers, J. W. , K. Büttgen , F. Leissing , et al. 2022. “An Advanced Method for the Release, Enrichment and Purification of High‐Quality *Arabidopsis thaliana* Rosette Leaf Trichomes Enables Profound Insights Into the Trichome Proteome.” Plant Methods 18, no. 1: 12. 10.1186/s13007-021-00836-0. 35086542 PMC8796501

[pce15357-bib-0039] Huebbers, J. W. , M. Mantz , R. Panstruga , and P. F. Huesgen . 2023. “Proteomics Dataset on Detached and Purified *Arabidopsis thaliana* Rosette Leaf Trichomes.” Data in Brief 46: 108897. 10.1016/j.dib.2023.108897. 36817732 PMC9936375

[pce15357-bib-0040] Hwang, I. S. , D. S. Choi , N. H. Kim , D. S. Kim , and B. K. Hwang . 2014. “The Pepper Cysteine/Histidine‐Rich DC1 Domain Protein CaDC1 Binds Both RNA and DNA and Is Required for Plant Cell Death and Defense Response.” New Phytologist 201, no. 2: 518–530. 10.1111/nph.12521. 24117868

[pce15357-bib-0041] Ichino, T. , K. Fuji , H. Ueda , et al. 2014. “GFS9/TT9 Contributes to Intracellular Membrane Trafficking and Flavonoid Accumulation in *Arabidopsis thaliana* .” Plant Journal 80, no. 3: 410–423. 10.1111/tpj.12637. 25116949

[pce15357-bib-0042] Ichino, T. , K. Maeda , I. Hara‐Nishimura , and T. Shimada . 2020. “Arabidopsis Echidna Protein Is Involved in Seed Coloration, Protein Trafficking to Vacuoles, and Vacuolar Biogenesis.” Journal of Experimental Botany 71, no. 14: 3999–4009. 10.1093/jxb/eraa147. 32201898 PMC7475254

[pce15357-bib-0043] Igisch, C. P. , C. Miège , and Y. Jaillais . 2022. “Cell Shape: A ROP Regulatory Tug‐of‐War in Pavement Cell Morphogenesis.” Current Biology 32, no. 3: R116–R118. 10.1016/j.cub.2021.12.028. 35134356

[pce15357-bib-0044] Ilgenfritz, H. , D. Bouyer , A. Schnittger , et al. 2003. “The Arabidopsis STICHEL Gene Is a Regulator of Trichome Branch Number and Encodes a Novel Protein.” Plant Physiology 131, no. 2: 643–655. 10.1104/pp.014209. 12586888 PMC166840

[pce15357-bib-0045] Jakoby, M. J. , D. Falkenhan , M. T. Mader , et al. 2008. “Transcriptional Profiling of Mature Arabidopsis Trichomes Reveals That NOECK Encodes the MIXTA‐Like Transcriptional Regulator Myb106.” Plant Physiology 148, no. 3: 1583–1602. 10.1104/pp.108.126979. 18805951 PMC2577251

[pce15357-bib-0046] Jin, S. , S. Zhang , Y. Liu , et al. 2020. “A Combination of Genome‐Wide Association Study and Transcriptome Analysis in Leaf Epidermis Identifies Candidate Genes Involved in Cuticular Wax Biosynthesis in *Brassica napus* .” BMC Plant Biology 20, no. 1: 458. 10.1186/s12870-020-02675-y. 33023503 PMC7541215

[pce15357-bib-0047] Kannangara, R. , C. Branigan , Y. Liu , et al. 2007. “The Transcription Factor WIN1/SHN1 Regulates Cutin Biosynthesis in *Arabidopsis thaliana* .” Plant Cell 19, no. 4: 1278–1294. 10.1105/tpc.106.047076. 17449808 PMC1913754

[pce15357-bib-0048] Kim, H. , H. Kwon , S. Kim , et al. 2016. “Synaptotagmin 1 Negatively Controls the Two Distinct Immune Secretory Pathways to Powdery Mildew Fungi in Arabidopsis.” Plant & Cell Physiology 57, no. 6: 1133–1141. 10.1093/pcp/pcw061. 27016097

[pce15357-bib-0049] Kollárová, E. , A. Baquero Forero , and F. Cvrčková . 2021. “The *Arabidopsis thaliana* Class Ii Formin FH13 Modulates Pollen Tube Growth.” Frontiers in Plant Science 12: 599961. 10.3389/fpls.2021.599961. 33679824 PMC7929981

[pce15357-bib-0050] Kriticos, D. J. , B. L. Webber , A. Leriche , et al. 2012. “Climond: Global High‐Resolution Historical and Future Scenario Climate Surfaces for Bioclimatic Modelling.” Methods in Ecology and Evolution 3, no. 1: 53–64. 10.1111/j.2041-210X.2011.00134.x.

[pce15357-bib-0051] Kulich, I. , Z. Vojtíková , M. Glanc , J. Ortmannová , S. Rasmann , and V. Žárský . 2015. “Cell Wall Maturation of Arabidopsis Trichomes Is Dependent on Exocyst Subunit EXO70H4 and Involves Callose Deposition.” Plant Physiology 168, no. 1: 120–131. 10.1104/pp.15.00112. 25767057 PMC4424025

[pce15357-bib-0052] Kulich, I. , Z. Vojtíková , P. Sabol , et al. 2018. “Exocyst Subunit EXO70H4 Has a Specific Role in Callose Synthase Secretion and Silica Accumulation.” Plant Physiology 176, no. 3: 2040–2051. 10.1104/pp.17.01693. 29301954 PMC5841730

[pce15357-bib-0053] Kuroda, H. , N. Takahashi , H. Shimada , M. Seki , K. Shinozaki , and M. Matsui . 2002. “Classification and Expression Analysis of Arabidopsis F‐Box‐Containing Protein Genes.” Plant & Cell Physiology 43, no. 10: 1073–1085. 10.1093/pcp/pcf151. 12407186

[pce15357-bib-0054] Larsen, P. B. , J. Cancel , M. Rounds , and V. Ochoa . 2007. “Arabidopsis ALS1 Encodes a Root Tip and Stele Localized Half Type ABC Transporter Required for Root Growth in an Aluminum Toxic Environment.” Planta 225, no. 6: 1447–1458. 10.1007/s00425-006-0452-4. 17171374

[pce15357-bib-0055] Lee, J. H. , and T. T. Paull . 2021. “Cellular Functions of the Protein Kinase Atm and Their Relevance to Human Disease.” Nature Reviews Molecular Cell Biology 22, no. 12: 796–814. 10.1038/s41580-021-00394-2. 34429537

[pce15357-bib-0056] Leonhardt, N. , J. M. Kwak , N. Robert , D. Waner , G. Leonhardt , and J. I. Schroeder . 2004. “Microarray Expression Analyses of Arabidopsis Guard Cells and Isolation of a Recessive Abscisic Acid Hypersensitive Protein Phosphatase 2C Mutant[W].” Plant Cell 16, no. 3: 596–615. 10.1105/tpc.019000. 14973164 PMC385275

[pce15357-bib-0057] Levy, A. , J. Y. Zheng , and S. G. Lazarowitz . 2015. “Synaptotagmin SYTA Forms ER‐Plasma Membrane Junctions That Are Recruited to Plasmodesmata for Plant Virus Movement.” Current Biology 25, no. 15: 2018–2025. 10.1016/j.cub.2015.06.015. 26166780 PMC4526382

[pce15357-bib-0058] Li, Y. , Y. Shen , C. Cai , et al. 2010. “The Type II Arabidopsis formin14 Interacts With Microtubules and Microfilaments to Regulate Cell Division.” Plant Cell 22, no. 8: 2710–2726. 10.1105/tpc.110.075507. 20709814 PMC2947165

[pce15357-bib-0059] Li, Z. , P. Wang , C. You , et al. 2020. “Combined GWAS and eQTL Analysis Uncovers a Genetic Regulatory Network Orchestrating the Initiation of Secondary Cell Wall Development in Cotton.” New Phytologist 226, no. 6: 1738–1752. 10.1111/nph.16468. 32017125

[pce15357-bib-0060] Lin, W. , and Z. Yang . 2020. “Unlocking the Mechanisms Behind the Formation of Interlocking Pavement Cells.” Current Opinion in Plant Biology 57: 142–154. 10.1016/j.pbi.2020.09.002. 33128897

[pce15357-bib-0061] Liu, E. , C. P. MacMillan , T. Shafee , et al. 2020. “Fasciclin‐Like Arabinogalactan‐Protein 16 (FLA16) Is Required for Stem Development in *Arabidopsis* .” Frontiers in Plant Science 11: 615392. 10.3389/fpls.2020.615392. 33362841 PMC7758453

[pce15357-bib-0062] Liu, S. , J. Jiao , T. J. Lu , F. Xu , B. G. Pickard , and G. M. Genin . 2017. “Arabidopsis Leaf Trichomes as Acoustic Antennae.” Biophysical Journal 113, no. 9: 2068–2076. 10.1016/j.bpj.2017.07.035. 29117529 PMC5685652

[pce15357-bib-0063] Liu, S. , F. Jobert , Z. Rahneshan , S. M. Doyle , and S. Robert . 2021. “Solving the Puzzle of Shape Regulation in Plant Epidermal Pavement Cells.” Annual Review of Plant Biology 72: 525–550. 10.1146/annurev-arplant-080720-081920. 34143651

[pce15357-bib-0064] Long, Z. , M. Tu , Y. Xu , et al. 2023. “Genome‐Wide‐Association Study and Transcriptome Analysis Reveal the Genetic Basis Controlling the Formation of Leaf Wax in *Brassica napus* .” Journal of Experimental Botany 74, no. 8: 2726–2739. 10.1093/jxb/erad047. 36724105

[pce15357-bib-0065] Mahjoob, M. M. M. , N. M. Kamal , Y. S. A. Gorafi , and H. Tsujimoto . 2022. “Genome‐Wide Association Study Reveals Distinct Genetic Associations Related to Leaf Hair Density in Two Lineages of Wheat‐Wild Relative *Aegilops tauschii* .” Scientific Reports 12, no. 1: 17486. 10.1038/s41598-022-21713-3. 36261481 PMC9581923

[pce15357-bib-0066] Mathur, J. , P. Spielhofer , B. Kost , and N. H. Chua . 1999. “The Actin Cytoskeleton Is Required to Elaborate and Maintain Spatial Patterning During Trichome Cell Morphogenesis in *Arabidopsis thaliana* .” Development 126, no. 24: 5559–5568. 10.1242/dev.126.24.5559. 10572033

[pce15357-bib-0067] Mitsuda, N. , K. Enami , M. Nakata , K. Takeyasu , and M. H. Sato . 2001. “Novel Type *Arabidopsis thaliana* H(+)‐PPase Is Localized to the Golgi Apparatus.” FEBS Letters 488, no. 1–2: 29–33. 10.1016/s0014-5793(00)02400-5. 11163790

[pce15357-bib-0068] Oulehlová, D. , E. Kollárová , P. Cifrová , P. Pejchar , V. Žárský , and F. Cvrčková . 2019. “Arabidopsis Class I Formin FH1 Relocates Between Membrane Compartments During Root Cell Ontogeny and Associates With Plasmodesmata.” Plant & Cell Physiology 60, no. 8: 1855–1870. 10.1093/pcp/pcz102. 31135031

[pce15357-bib-0069] Parsons, H. T. , K. Christiansen , B. Knierim , et al. 2012. “Isolation and Proteomic Characterization of the Arabidopsis Golgi Defines Functional and Novel Components Involved in Plant Cell Wall Biosynthesis.” Plant Physiology 159, no. 1: 12–26. 10.1104/pp.111.193151. 22430844 PMC3375956

[pce15357-bib-0070] Pasha, A. , S. Subramaniam , A. Cleary , et al. 2020. “Araport Lives: An Updated Framework for Arabidopsis Bioinformatics.” Plant Cell 32, no. 9: 2683–2686. 10.1105/tpc.20.00358. 32699173 PMC7474289

[pce15357-bib-0071] Peco, J. D. , P. Higueras , J. A. Campos , A. Olmedilla , M. C. Romero‐Puertas , and L. M. Sandalio . 2020. “Deciphering Lead Tolerance Mechanisms in a Population of the Plant Species *Biscutella auriculata* L. From a Mining Area: Accumulation Strategies and Antioxidant Defenses.” Chemosphere 261: 127721. 10.1016/j.chemosphere.2020.127721. 32745740

[pce15357-bib-0072] Pietra, S. , A. Gustavsson , C. Kiefer , et al. 2013. “Arabidopsis SABRE and CLASP Interact to Stabilize Cell Division Plane Orientation and Planar Polarity.” Nature Communications 4: 2779. 10.1038/ncomms3779. PMC3868209 24240534

[pce15357-bib-0073] Python Software Foundation . 2024. “Python.” https://www.python.org/.

[pce15357-bib-0074] Python Visualisation . 2024. “Folium.” https://python-visualization.github.io/folium/latest/.

[pce15357-bib-0075] Qu, J. , D. Bonte , and M. L. Vandegehuchte . 2022. “Phenotypic and Genotypic Divergence of Plant‐Herbivore Interactions Along an Urbanization Gradient.” Evolutionary Applications 15, no. 5: 865–877. 10.1111/eva.13376. 35603025 PMC9108311

[pce15357-bib-0076] Radua, J. , A. Albajes‐Elsagirre , and L. Fortea 2024. “FDR Online Calculator.” Seed‐Based *d* Mapping. https://www.sdmproject.com/utilities/?show=FDR.

[pce15357-bib-0077] Reback, J. , jbrockmendel , W. McKinney , et al. 2021. “Pandas‐Dev/Pandas: Pandas 1.3.5 (v. 1.3.5).” Zenodo. 10.5281/zenodo.5774815.

[pce15357-bib-0078] Reiser, L. , E. Bakker , S. Subramaniam , et al. 2024. “The Arabidopsis Information Resource in 2024.” Genetics 227, no. 1: iyae027. 10.1093/genetics/iyae027. 38457127 PMC11075553

[pce15357-bib-0079] Reynoud, N. , J. Petit , C. Bres , et al. 2021. “The Complex Architecture of Plant Cuticles and Its Relation to Multiple Biological Functions.” Frontiers in Plant Science 12: 782773. 10.3389/fpls.2021.782773. 34956280 PMC8702516

[pce15357-bib-0080] Ricachenevsky, F. K. , T. Punshon , D. E. Salt , J. P. Fett , and M. L. Guerinot . 2021. “ *Arabidopsis thaliana* Zinc Accumulation in Leaf Trichomes Is Correlated With Zinc Concentration in Leaves.” Scientific Reports 11, no. 1: 5278. 10.1038/s41598-021-84508-y. 33674630 PMC7935932

[pce15357-bib-0081] Ristova, D. , M. Giovannetti , K. Metesch , and W. Busch . 2018. “Natural Genetic Variation Shapes Root System Responses to Phytohormones in Arabidopsis.” Plant Journal: for Cell and Molecular Biology 96, no. 2: 468–481. 10.1111/tpj.14034. 30030851 PMC6220887

[pce15357-bib-0082] Rosero, A. , D. Oulehlová , L. Stillerová , et al. 2016. “Arabidopsis FH1 Formin Affects Cotyledon Pavement Cell Shape by Modulating Cytoskeleton Dynamics.” Plant & Cell Physiology 57, no. 3: 488–504. 10.1093/pcp/pcv209. 26738547

[pce15357-bib-0083] Rosero, A. , V. Žárský , and F. Cvrčková . 2013. “AtFH1 Formin Mutation Affects Actin Filament and Microtubule Dynamics in *Arabidopsis thaliana* .” Journal of Experimental Botany 64, no. 2: 585–597. 10.1093/jxb/ers351. 23202131 PMC3542049

[pce15357-bib-0084] Sapala, A. , A. Runions , A. L. Routier‐Kierzkowska , et al. 2018. “Why Plants Make Puzzle Cells, and How Their Shape Emerges.” eLife 7: e32794. 10.7554/eLife.32794. 29482719 PMC5841943

[pce15357-bib-0085] Sato, Y. , R. Shimizu‐Inatsugi , M. Yamazaki , K. K. Shimizu , and A. J. Nagano . 2019. “Plant Trichomes and a Single Gene GLABRA1 Contribute to Insect Community Composition on Field‐Grown *Arabidopsis thaliana* .” BMC Plant Biology 19, no. 1: 163. 10.1186/s12870-019-1705-2. 31029092 PMC6486987

[pce15357-bib-0086] Schapire, A. L. , B. Voigt , J. Jasik , et al. 2008. “Arabidopsis Synaptotagmin 1 Is Required for the Maintenance of Plasma Membrane Integrity and Cell Viability.” Plant Cell 20, no. 12: 3374–3388. 10.1105/tpc.108.063859. 19088329 PMC2630439

[pce15357-bib-0087] Schindelin, J. , I. Arganda‐Carreras , E. Frise , et al. 2012. “Fiji: An Open‐Source Platform for Biological‐Image Analysis.” Nature Methods 9, no. 7: 676–682. 10.1038/nmeth.2019. 22743772 PMC3855844

[pce15357-bib-0088] Schober, P. , C. Boer , and L. A. Schwarte . 2018. “Correlation Coefficients: Appropriate Use and Interpretation.” Anesthesia & Analgesia 126, no. 5: 1763–1768. 10.1213/ANE.0000000000002864. 29481436

[pce15357-bib-0089] Scutenaire, J. , J. M. Deragon , V. Jean , et al. 2018. “The Yth Domain Protein ECT2 Is an M^6^A Reader Required for Normal Trichome Branching in Arabidopsis.” Plant Cell 30, no. 5: 986–1005. 10.1105/tpc.17.00854. 29618631 PMC6002185

[pce15357-bib-0090] Seregin, I. V. , and V. B. Ivanov . 1997. “Histochemical Investigation of Cadmium and Lead Distribution in Plants.” Russian Journal of Plant Physiology 44, no. 6: 791–796. https://www.researchgate.net/publication/279705125_Histochemical_Investigation_of_Cadmium_and_Lead_Distribution_in_Plants.

[pce15357-bib-0091] Seren, Ü. 2015. “The GWA‐Portal Resource for Phenotypes and GWAS Studies.” https://gwas.gmi.oeaw.ac.at/.

[pce15357-bib-0092] Seren, Ü. , B. J. Vilhjálmsson , M. W. Horton , et al. 2012. “GWAPP: A Web Application for Genome‐Wide Association Mapping in Arabidopsis.” Plant Cell 24, no. 12: 4793–4805. 10.1105/tpc.112.108068. 23277364 PMC3556958

[pce15357-bib-0093] Shi, C. , and H. Liu . 2021. “How Plants Protect Themselves From Ultraviolet‐B Radiation Stress.” Plant Physiology 187, no. 3: 1096–1103. 10.1093/plphys/kiab245. 34734275 PMC8566272

[pce15357-bib-0094] Slovak, R. , C. Göschl , Ü. Seren , and W. Busch . 2015. “Genome‐Wide Association Mapping in Plants Exemplified for Root Growth in *Arabidopsis thaliana* .” Methods in Molecular Biology 1284: 343–357. 10.1007/978-1-4939-2444-8_17. 25757781

[pce15357-bib-0095] Srivastava, R. K. , P. Pandey , R. Rajpoot , A. Rani , and R. S. Dubey . 2014. “Cadmium and Lead Interactive Effects on Oxidative Stress and Antioxidative Responses in Rice Seedlings.” Protoplasma 251, no. 5: 1047–1065. 10.1007/s00709-014-0614-3. 24482190

[pce15357-bib-0096] Stagroom, J. 2024. “Chi‐Square Test Calculator.” Social Science Statistics. https://www.socscistatistics.com/tests/chisquare2/default2.aspx.

[pce15357-bib-0097] Suh, M. C. , A. L. Samuels , R. Jetter , et al. 2005. “Cuticular Lipid Composition, Surface Structure, and Gene Expression in Arabidopsis Stem Epidermis.” Plant Physiology 139, no. 4: 1649–1665. 10.1104/pp.105.070805. 16299169 PMC1310549

[pce15357-bib-0098] Symonds, V. V. , G. Hatlestad , and A. M. Lloyd . 2011. “Natural Allelic Variation Defines a Role for ATMYC1: Trichome Cell Fate Determination.” PLoS Genetics 7, no. 6: e1002069. 10.1371/journal.pgen.1002069. 21695236 PMC3111535

[pce15357-bib-0099] Szklarczyk, D. , R. Kirsch , M. Koutrouli , et al. 2023. “The STRING Database in 2023: Protein‐Protein Association Networks and Functional Enrichment Analyses for Any Sequenced Genome of Interest.” Nucleic Acids Research 51, no. D1: D638–D646. 10.1093/nar/gkac1000. 36370105 PMC9825434

[pce15357-bib-0100] Szymanski, D. B. 2005. “Breaking the WAVE Complex: The Point of Arabidopsis Trichomes.” Current Opinion in Plant Biology 8, no. 1: 103–112. 10.1016/j.pbi.2004.11.004. 15653407

[pce15357-bib-0101] Togninalli, M. , Ü. Seren , J. A. Freudenthal , et al. 2020. “Arapheno and the AraGWAS Catalog 2020: A Major Database Update Including RNA‐Seq and Knockout Mutation Data for *Arabidopsis thaliana* .” Nucleic Acids Research 48, no. D1: 1063. 10.1093/nar/gkz925. PMC7145550 31642487

[pce15357-bib-0102] Torii, K. U. 2021. “Stomatal Development in the Context of Epidermal Tissues.” Annals of Botany 128, no. 2: 137–148. 10.1093/aob/mcab052. 33877316 PMC8324025

[pce15357-bib-0103] Vasavada, N. 2016. “Fisher Test of Exact Count Data.” Online Web Statistical Calculators. https://astatsa.com/FisherTest.

[pce15357-bib-0104] Vega‐Muñoz, I. , A. Herrera‐Estrella , O. Martínez‐de la Vega , and M. Heil . 2023. “ATM and ATR, Two Central Players of the Dna Damage Response, Are Involved in the Induction of Systemic Acquired Resistance by Extracellular DNA, but Not the Plant Wound Response.” Frontiers in Immunology 14: 1175786. 10.3389/fimmu.2023.1175786. 37256140 PMC10225592

[pce15357-bib-0105] Vukašinović, N. , and V. Žárský . 2016. “Tethering Complexes in the Arabidopsis Endomembrane System.” Frontiers in Cell and Developmental Biology 4: 46. 10.3389/fcell.2016.00046. 27243010 PMC4871884

[pce15357-bib-0106] Wang, X. , Y. Miao , Y. Cai , et al. 2021. “Large‐Fragment Insertion Activates Gene Gafz (Ga08G0121) and Is Associated With the Fuzz and Trichome Reduction in Cotton (*Gossypium arboreum*).” Plant Biotechnology Journal 19, no. 6: 1110–1124. 10.1111/pbi.13532. 33369825 PMC8196653

[pce15357-bib-0107] Waskom, M. 2021. “Seaborn: Statistical Data Visualization.” Journal of Open Source Software 6, no. 60: 3021. 10.21105/joss.03021.

[pce15357-bib-0108] Wei, L. H. , P. Song , Y. Wang , et al. 2018. “The M^6^A Reader ECT2 Controls Trichome Morphology by Affecting mRNA Stability in Arabidopsis.” Plant Cell 30, no. 5: 968–985. 10.1105/tpc.17.00934. 29716990 PMC6002187

[pce15357-bib-0109] Wienkoop, S. , D. Zoeller , B. Ebert , et al. 2004. “Cell‐Specific Protein Profiling in *Arabidopsis thaliana* Trichomes: Identification of Trichome‐Located Proteins Involved in Sulfur Metabolism and Detoxification.” Phytochemistry 65, no. 11: 1641–1649. 10.1016/j.phytochem.2004.03.026. 15276459

[pce15357-bib-0110] Wu, Y. , Q. Xun , Y. Guo , et al. 2016. “Genome‐Wide Expression Pattern Analyses of the Arabidopsis Leucine‐Rich Repeat Receptor‐Like Kinases.” Molecular Plant 9, no. 2: 289–300. 10.1016/j.molp.2015.12.011. 26712505

[pce15357-bib-0111] Xia, K. , H. X. Sun , J. Li , et al. 2022. “The Single‐Cell Stereo‐Seq Reveals Region‐Specific Cell Subtypes and Transcriptome Profiling in Arabidopsis Leaves.” Developmental Cell 57, no. 10: 1299–1310.e4. 10.1016/j.devcel.2022.04.011. 35512702

[pce15357-bib-0112] Xu, J. J. , X. Fang , C. Y. Li , et al. 2018. “Characterization of *Arabidopsis thaliana* Hydroxyphenylpyruvate Reductases in the Tyrosine Conversion Pathway.” Frontiers in Plant Science 9: 1305. 10.3389/fpls.2018.01305. 30233632 PMC6133988

[pce15357-bib-0113] Xuan, L. , T. Yan , L. Lu , et al. 2020. “Genome‐Wide Association Study Reveals New Genes Involved in Leaf Trichome Formation in Polyploid Oilseed Rape (*Brassica napus* L.).” Plant, Cell & Environment 43, no. 3: 675–691. 10.1111/pce.13694. 31889328

[pce15357-bib-0114] Yaashikaa, P. R. , P. S. Kumar , S. Jeevanantham , and R. Saravanan . 2022. “A Review on Bioremediation Approach for Heavy Metal Detoxification and Accumulation in Plants.” Environmental Pollution 301: 119035. 10.1016/j.envpol.2022.119035. 35196562

[pce15357-bib-0115] Yamazaki, T. , Y. Kawamura , A. Minami , and M. Uemura . 2008. “Calcium‐Dependent Freezing Tolerance in Arabidopsis Involves Membrane Resealing via Synaptotagmin SYT1.” Plant Cell 20, no. 12: 3389–3404. 10.1105/tpc.108.062679. 19088330 PMC2630449

[pce15357-bib-0116] Yan, H. , D. C. Haak , S. Li , L. Huang , and A. Bombarely . 2022. “Exploring Transposable Element‐Based Markers to Identify Allelic Variations Underlying Agronomic Traits in Rice.” Plant Communications 3, no. 3: 100270. 10.1016/j.xplc.2021.100270. 35576152 PMC9251385

[pce15357-bib-0117] Yates, A. D. , J. Allen , R. M. Amode , et al. 2022. “Ensembl Genomes 2022: An Expanding Genome Resource for Non‐Vertebrates.” Nucleic Acids Research 50, no. D1: D996–D1003. 10.1093/nar/gkab1007. 34791415 PMC8728113

[pce15357-bib-0118] Yengo, L. , S. Vedantam , E. Marouli , et al. 2022. “A Saturated Map of Common Genetic Variants Associated With Human Height.” Nature 610, no. 7933: 704–712. 10.1038/s41586-022-05275-y. 36224396 PMC9605867

[pce15357-bib-0119] Yuan, D. S. 2011. “Dithizone Staining of Intracellular Zinc: An Unexpected and Versatile Counterscreen for Auxotrophic Marker Genes in *Saccharomyces cerevisiae* .” PLoS One 6, no. 10: e25830. 10.1371/journal.pone.0025830. 21998704 PMC3187812

[pce15357-bib-0120] Zeng, L. , T. Zhu , Y. Gao , et al. 2017. “Effects of Ca Addition on the Uptake, Translocation, and Distribution of Cd in *Arabidopsis thaliana* .” Ecotoxicology and Environmental Safety 139: 228–237. 10.1016/j.ecoenv.2017.01.023. 28152404

[pce15357-bib-0121] Zhang, D. 2009. “Homology between DUF784, DUF1278 Domains and the Plant Prolamin Superfamily Typifies Evolutionary Changes of Disulfide Bonding Patterns.” Cell Cycle 8, no. 20: 3428–3430. 10.4161/cc.8.20.9674. 19806031

[pce15357-bib-0122] Zhao, Z. , W. Zhang , B. A. Stanley , and S. M. Assmann . 2008. “Functional Proteomics of *Arabidopsis thaliana* Guard Cells Uncovers New Stomatal Signaling Pathways.” Plant Cell 20, no. 12: 3210–3226. 10.1105/tpc.108.063263. 19114538 PMC2630442

[pce15357-bib-0123] Zhou, L. H. , S. B. Liu , P. F. Wang , et al. 2017. “The Arabidopsis Trichome Is an Active Mechanosensory Switch.” Plant, Cell & Environment 40, no. 5: 611–621. 10.1111/pce.12728. 26920667

[pce15357-bib-0124] Zuch, D. T. , S. M. Doyle , M. Majda , R. S. Smith , S. Robert , and K. U. Torii . 2022. “Cell Biology of the Leaf Epidermis: Fate Specification, Morphogenesis, and Coordination.” Plant Cell 34, no. 1: 209–227. 10.1093/plcell/koab250. 34623438 PMC8774078

