# Supplementary material for: A Genome‐Wide Association Screen for Genes Affecting Leaf Trichome Development and Epidermal Metal Accumulation in Arabidopsis
Source: Plant Cell Environ. 2025 Jan 15;48(5):3708–34. doi: 10.1111/pce.15357 (PMC11963502; doi:10.1111/pce.15357)
Supplement: Supplementary file 1 — Supporting information. [file PCE-48-3708-s001.pdf]

**Supplementary figures for Bezvoda et al.: A genome-wide association screen for genes affecting leaf trichome development and epidermal metal accumulation in Arabidopsis**

**Figure S1. Mutual correlation of all epidermal phenotype parameters analyzed.** Distribution of values for each parameter is shown at the diagonal of the matrix, with accession counts on the Y axis. Spearman's correlation coefficients (Rho), shown in the upper right triangle for all parameter pairs, are color-coded: gray for negligible correlation ($-0.1 < Rho < 0.1$), magenta for positive, cyan for negative correlation, with intensity of coloring reflecting correlation strength, while P-values for these correlations, shown in the lower left triangle, are color-coded from green (highly significant, $0 < P \leq 0.01$ with darker color corresponding to lower P) through yellow (significant, $0.01 < P \leq 0.05$) to orange ($P > 0.05$, not significant).

**Figure S2. Distribution of SNP types identified as significantly associated with individual parameters**. Only traits with detected polymorphisms in more than 5 loci are shown.

**Figure S3. Geographic allele distribution for selected genes with significantly environmentally correlated polymorphism.** Sites of origin of accessions harboring the reference allele are shown in blue, those with the minor allele in red (for allele description see Table 4, Table 6 and Figure 7A).

**Figure S4. Distribution of all standard climatic variable values for the reference and quintuple substitution minor allele of GFS9/TT9.** Data for whole Ensembl accessions set are shown. Accessions where some of the SNPs characteristic for the quintuple allele are missing due to sequencing gaps ("probable quintuple") are included in the quintuple mutant category. FDR-corrected P-values for the observed differences are color-coded green for highly significant, $0 < P \leq 0.01$ (with darker color corresponding to lower P), yellow for significant ($0.01 < P \leq 0.05$), and orange for not significant ($P > 0.05$). Red lines indicate median values.

**Figure S5. A detailed map of predicted associations among candidate genes for individual trait groups.** Thickness of graph edges reflects strength of evidence. Numbers correspond to the numbering of gene clusters in Supplementary Table S10.

**Figure S6. Inter-observer replicability of selected parameters estimation**. For continuous variables, results from two independent screens of the same images are plotted against each other (X-axis values correspond to averages of three plants). For categorical ordinate variables, the replicability values correspond to the percentage of accessions where results for at least two out the three plants differed by no more than 1 between two independent visual screens of the same images.

**Figure S1. Mutual correlation of all epidermal phenotype parameters analyzed.**

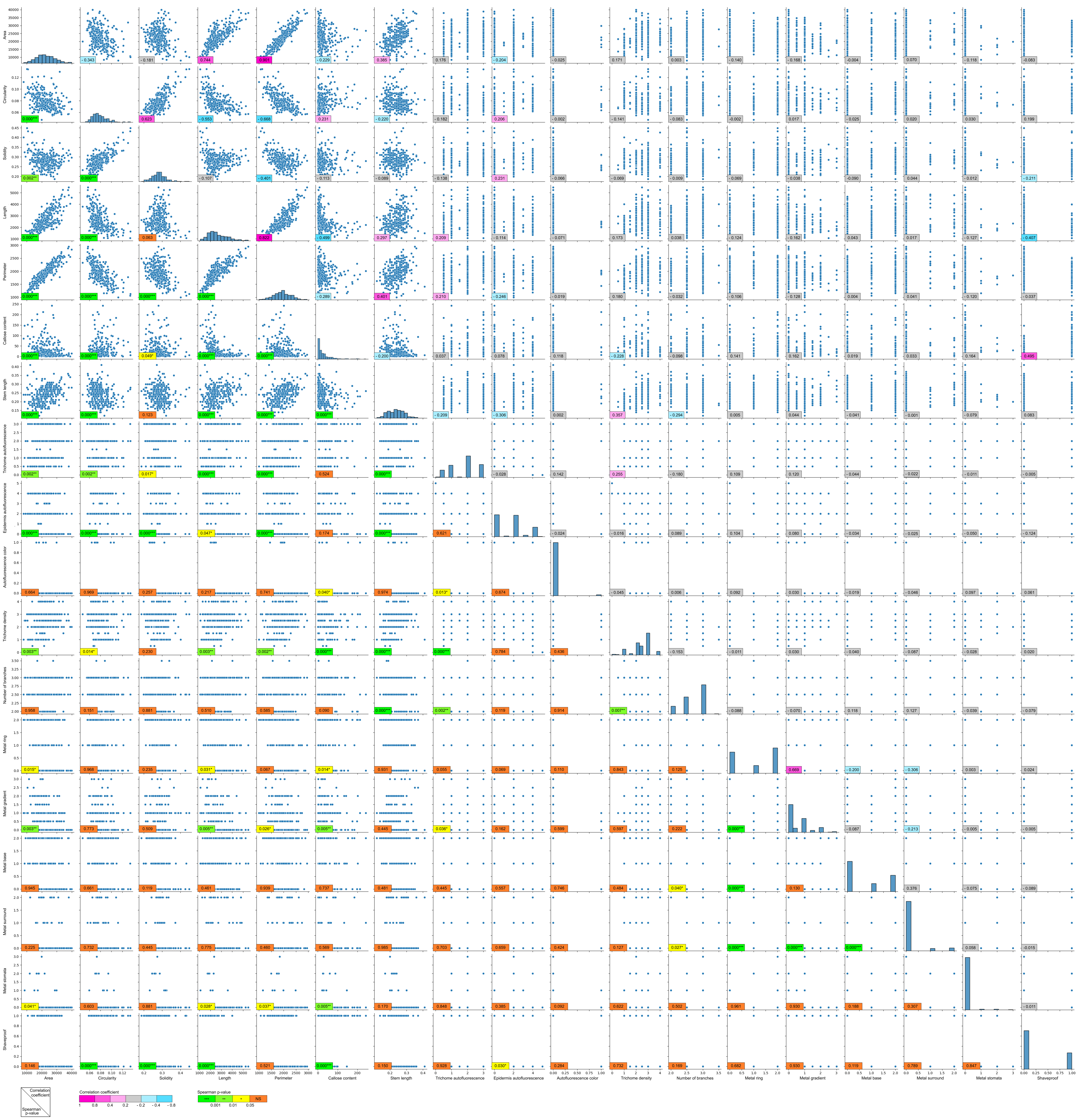

**Figure S2. Distribution of SNP types identified as significantly associated with individual parameters.**

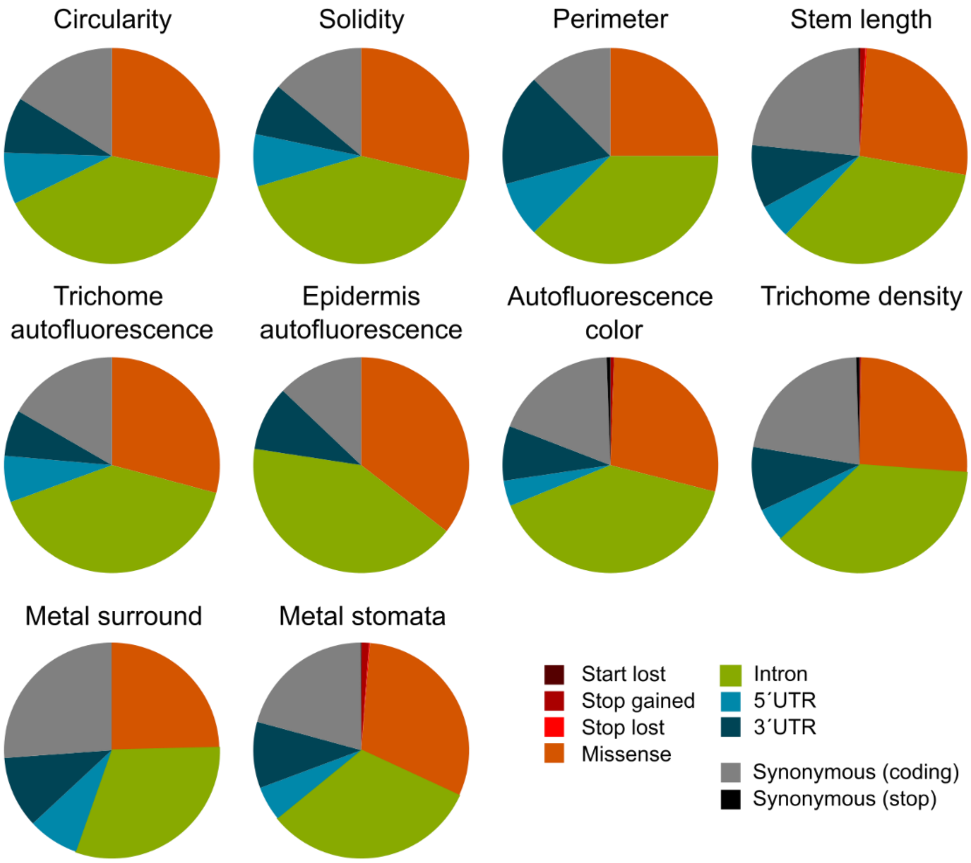

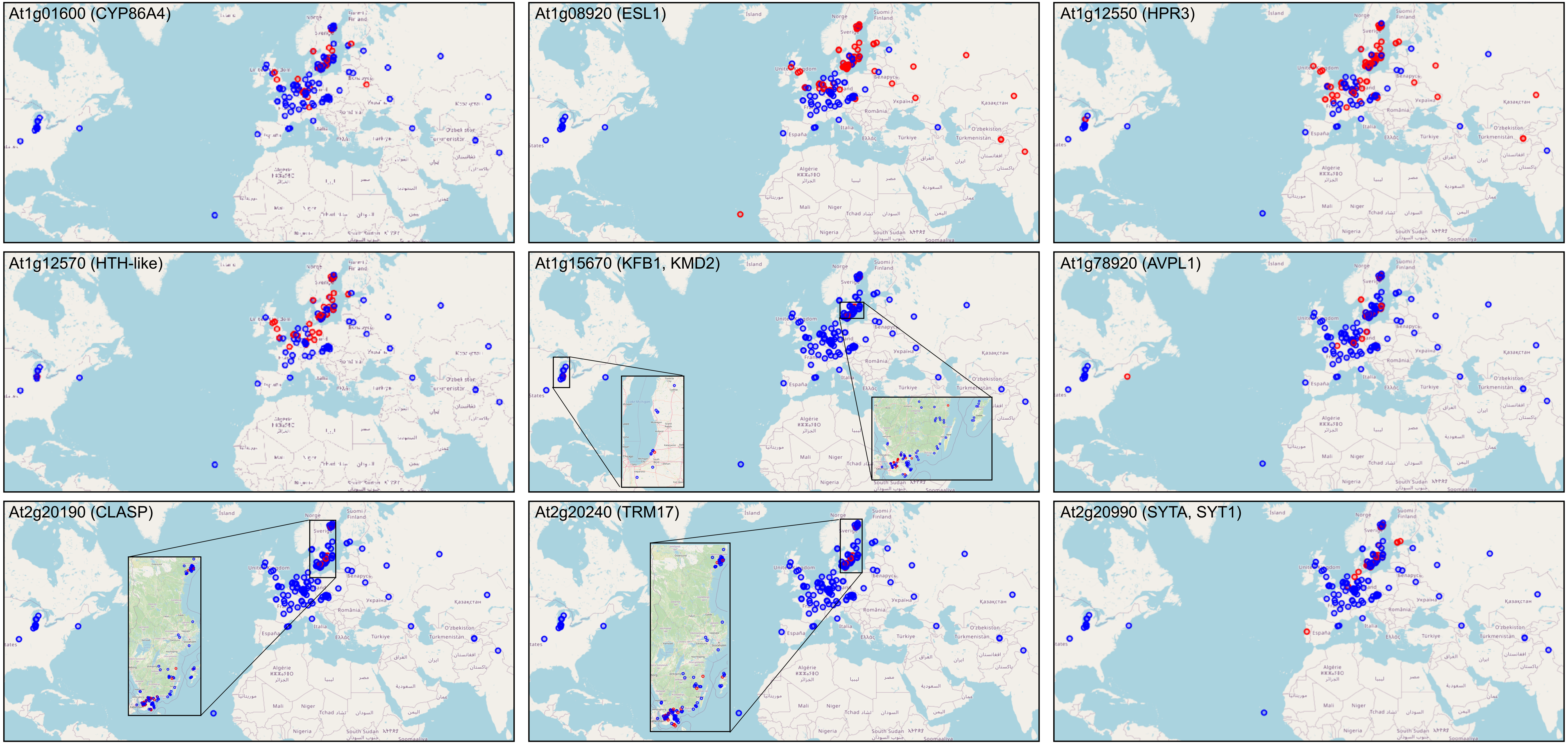

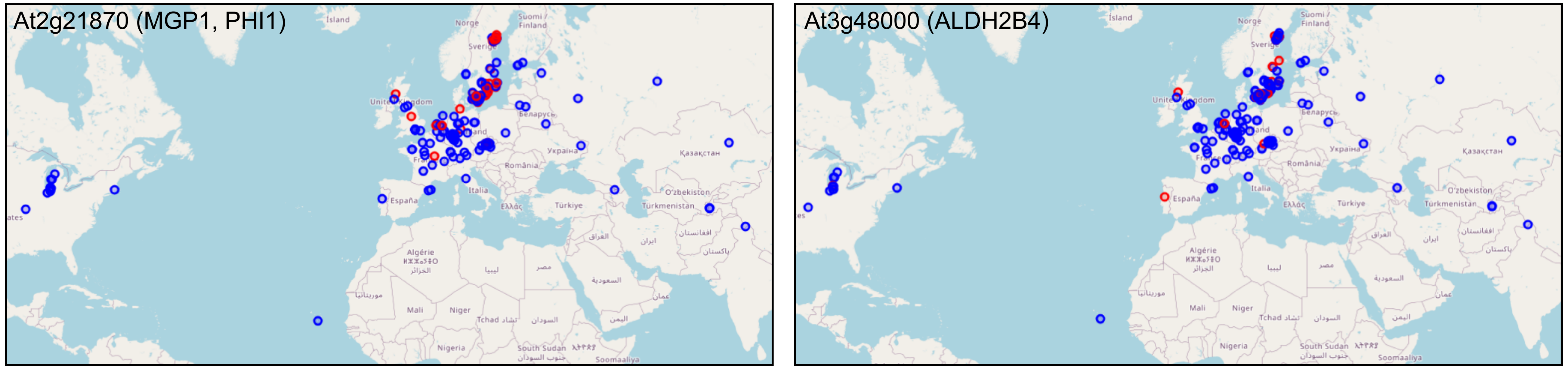


**Figure S3. Geographic allele distribution for selected genes with significantly environmentally correlated polymorphism.**

**Figure S4. Distribution of all standard climatic variable values for the reference and quintuple substitution minor allele of GFS9/TT9.**

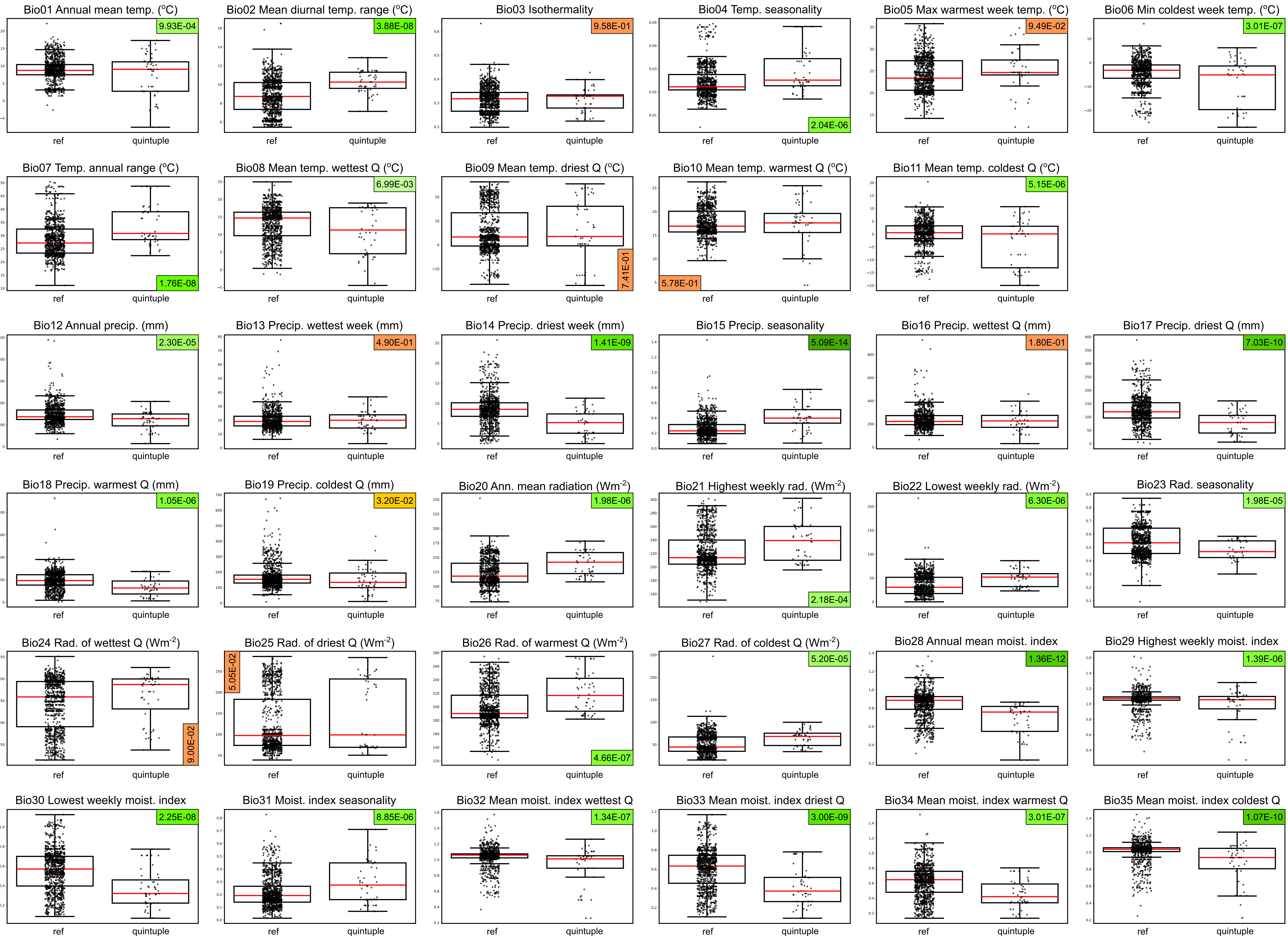

**Figure S5. A detailed map of predicted associations among candidate genes for individual trait groups.**

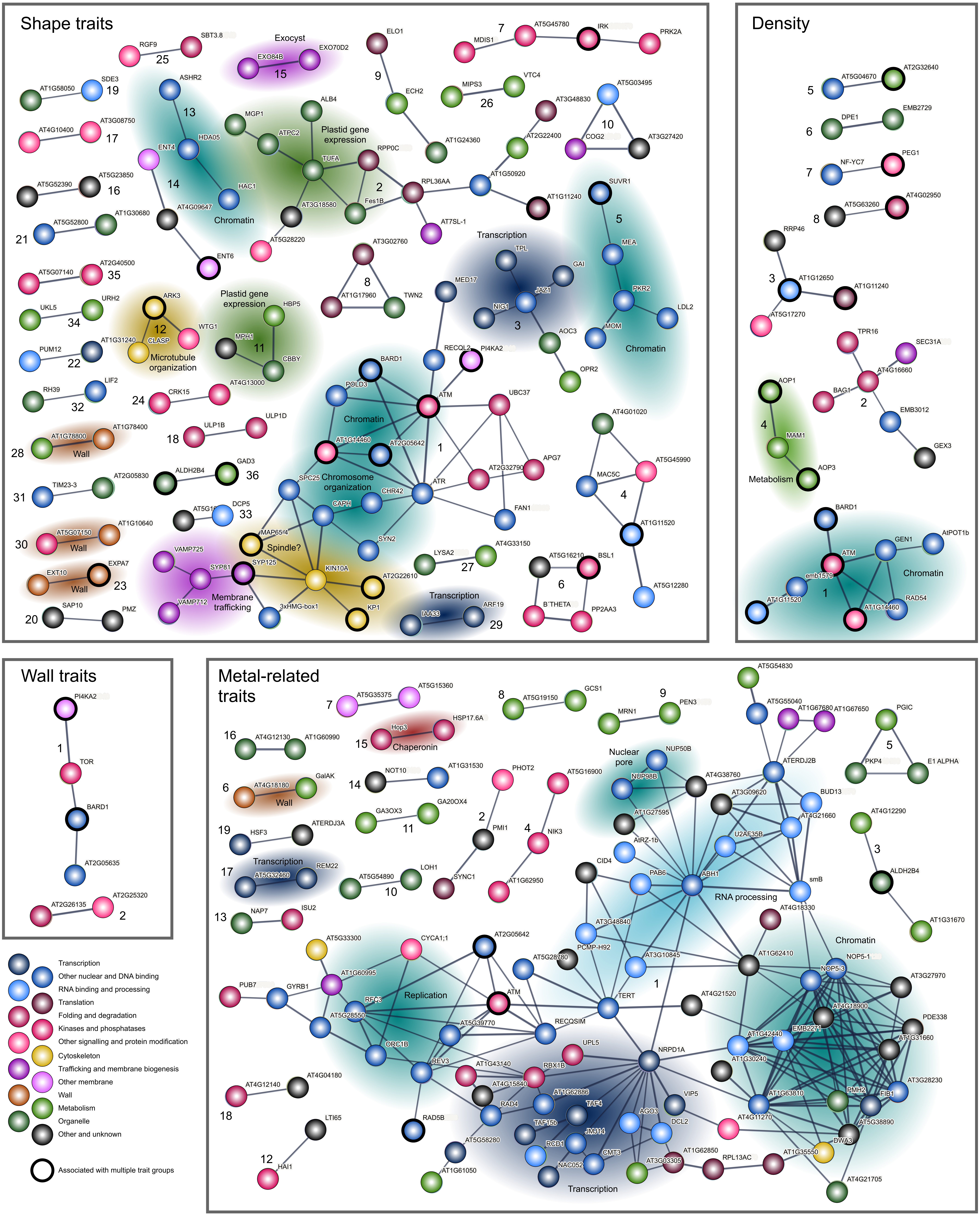

**Figure S6. Inter-observer replicability of selected parameters estimation**.

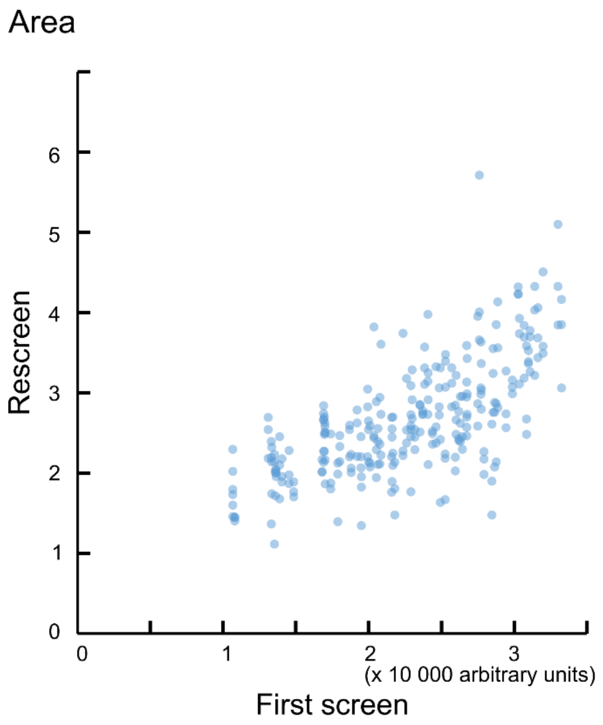


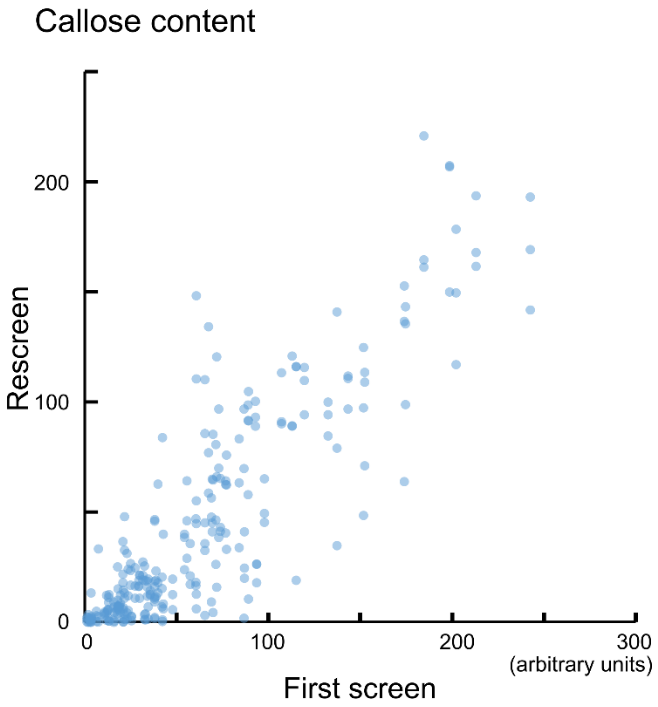


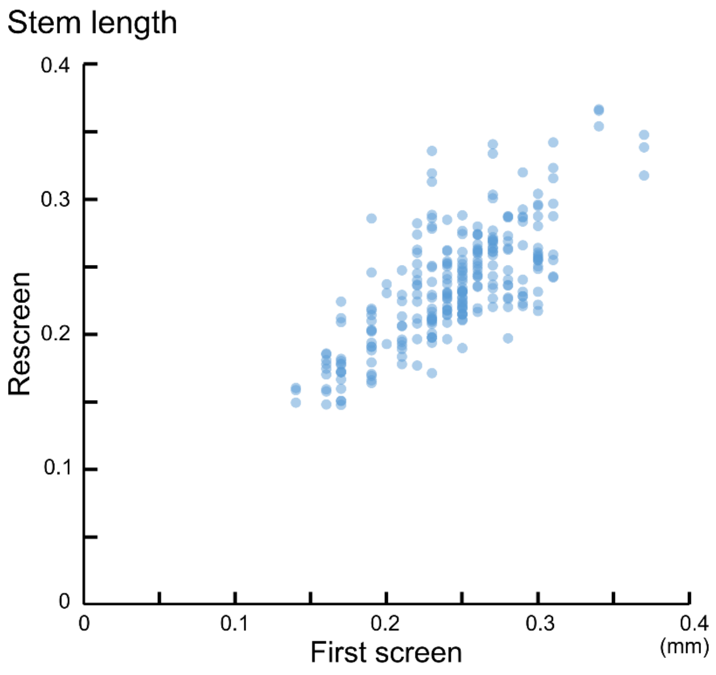


Categorical variables

| Parameter | Accessions rescreened | Replicability (%) |
|---|---|---|
| Trichome autofluorescence | 89 | 99 |
| Epidermis autofluorescence | 89 | 99 |
| Density | 90 | 98 |
| Number of branches | 89 | 97 |
| Metal ring | 88 | 100 |
| Metal gradient | 88 | 99 |
| Metal base | 88 | 98 |
| Metal surround | 88 | 95 |
| Metal stomata | 88 | 100 |
